# Current Evidence and Emerging Directions in Endometriosis Pharmacotherapy: A Systematic Review of Recent Advances

**DOI:** 10.3390/jcm15187238

**Published:** 2026-09-17

**Authors:** Zuzanna Betiuk, Martyna Błażyca, Martyna Bartoszczyk, Katarzyna Kutyła, Natalia Gierulska, Rafał Tarkowski, Krzysztof Kułak

**Affiliations:** 1 Students’ Scientific Association at the 1st Chair and Department of Oncological Gynecology and Gynecology, Medical University of Lublin, 20-081 Lublin, Poland; 2 1st Chair and Department of Oncological Gynaecology and Gynaecology, Medical University of Lublin, Staszica 16 St., 20-081 Lublin, Poland

**Keywords:** endometriosis, pharmacotherapy, immunotherapy, dienogest, GnRH antagonist

## Abstract

**Background**: Endometriosis affects 190 million women worldwide, so there is a need for accessible and more comprehensive knowledge regarding the pharmacotherapeutic options that can be offered to patients. This systematic review (2019–2025) synthesizes research results regarding the efficacy and safety of dienogest (DNG) and GnRH antagonists, while also summarizing research on new directions in endometriosis treatment. **Methods**: Following PRISMA 2020 guidelines, we searched Medline for clinical evidence on established standards and emerging therapies. A structured narrative synthesis was performed. Quality was assessed using Cochrane RoB 2 and ROBINS-I tools. **Results**: A total of 44 out of 926 records were analyzed. The review confirms the efficacy of DNG as a first-line treatment for endometriosis. Relugolix combination therapy appears to be a more effective and safer option than elagolix or relugolix monotherapy in reducing dysmenorrhea and pelvic pain. Research concerning immunotherapy for endometriosis (using tocilizumab, anakinra, bevacizumab, and anti-CD47 antibodies) is subject to a high risk of bias and very low GRADE certainty. Nevertheless, these findings may serve as a foundation for further studies, which are necessary to evaluate their efficacy and safety in the treatment of endometriosis. **Conclusions**: DNG is generally well-tolerated, but further studies are needed to unequivocally determine the impact of DNG on BMD loss and cancer risk. Research on GnRH antagonists indicates their increasing role in the future management of endometriosis. There are new perspectives in endometriosis pharmacotherapy—including immunotherapy; however, there is a fundamental need for their further development and evaluation.

## 1. Introduction

Endometriosis is a major issue in the field of gynecology since it affects almost 190 million women [1]. In the course of the disease, ectopic foci of estrogen-dependent endometrial-like tissue develop within the pelvic cavity and in extra pelvic locations. There are numerous types and locations of endometriosis: peritoneal (superficial), ovarian, deep, bowel or bladder endometriosis [2]. It is a chronic inflammatory disease which highly impacts daily life—the most common symptoms include non-menstrual pelvic pain (NMPP), dysmenorrhea, heavy menstrual bleeding, deep dyspareunia and infertility. Patients also struggle with symptoms from endometriosis lesions localized beyond the reproductive organs such as bowel dysfunction and urinary problems. The symptoms in patients should guide clinicians toward undertaking a diagnostic workup which consists of physical examination, imaging and laparoscopy with histopathological examination of the lesions. An advantage of laparoscopy as a gold standard is the ability to both assess endometrial lesions and simultaneously intervene by removing them, particularly when previous pharmacological treatments have proven ineffective [3]. The average time to diagnosis may range from 4 to 12 years, with the mean age of patients being approximately 33 years at that time [4]. A classification used in cases of endometriosis is the revised American Society for Reproductive Medicine (rASRM) classification. It is based on the size of lesions in the ovaries, fallopian tubes and peritoneum as well as the severity of adhesion. In cases of deep infiltrating endometriosis, the ENZIAN classification system is additionally employed to provide a detailed assessment of retroperitoneal structure. It also takes into account extragenital locations including ureteral and diaphragmatic lesions [5]. When it comes to deep endometriosis pain, symptoms may be particularly severe due to the anatomical distribution of endometriotic lesions, which frequently involve infiltration of the inferior hypogastric nerve [6]. Treatment of endometriosis consists of pharmacotherapy, as well as surgical management. Its purpose is to relieve pain and other symptoms without targeting infertility. Choosing a proper treatment depends on the severity of this condition, but also on a woman’s future reproductive plans [3].

The aim of the following article is to review the literature revolving around effective methods of pharmacology treatment, along with those beyond the actual pharmacotherapy recommendations.

The research question was formulated within criteria (PICO framework). The population included premenopausal women aged 15–50 years with diagnosis of endometriosis. There were also supplementary populations included for the purpose of preliminary assessment and narrative synthesis, such as healthy human volunteers and animal models. Used for narrative synthesis or research directions, they were excluded from the main synthesis of clinical efficacy and patient-reported outcomes. The review incorporated studies assessing the efficacy and safety of pharmacological interventions used in the treatment of endometriosis along with Progestins, GnRH Antagonists, GnRH Agonists, drug combinations and immunotherapy agents currently being explored for endometriosis treatment. Studies on immunotherapy were included for the purpose of a narrative synthesis on new research perspectives but were excluded from the main synthesis of clinical efficacy and patient-reported outcomes for approved standard-of-care agents. Comparisons or control groups included both non-active and active comparator arms: placebo, no treatment control groups or active control groups with any current standard pharmacological treatment for endometriosis. Some of the included studies were non-comparative (open-label extension studies, cohort studies) and were crucial for collecting long-term safety and adverse event data, or for providing exploratory data. The outcomes comprised endometriosis-associated pain, quality of life, endometriosis lesion size, comprehensive safety profile and exploratory markers (Table 1).

## 2. Methods

This systematic review was conducted in accordance with the Preferred Reporting Items for Systematic Reviews and Meta-Analyses (PRISMA) guidelines. The protocol for this systematic review was registered in the International Prospective Register of Systematic Reviews (PROSPERO) under the registration number CRD420251183131.

The Medline/PubMed database was searched and the results covered publications from 1 December 2019 to 1 December 2025. To ensure a comprehensive search and identify the gray literature, Google Scholar and trial registries, including ClinicalTrials.gov were searched. Handsearching of the reference lists of included studies was performed. The study selection process was conducted independently by four reviewers with disagreements resolved by consensus. The complete search strategy for Medline is provided in Supplementary Materials (Systematic Review Search Strategy).

Studies including premenopausal women aged 15–50 years with endometriosis were included. While the primary analysis focused on clinical efficacy in patients, supplementary populations were also included for exploratory synthesis. These included healthy human volunteers (to assess pharmacological profiles) and animal models (to explore mechanisms of action and early-stage efficacy). Data from these groups were narratively synthesized to identify future research directions.

The interventions of studies included pharmacological treatments, which were categorized as: hormonal therapies, novel agents (dopamine agonists and immunotherapy agents) and antioxidants. Immunotherapy was included for a narrative, exploratory synthesis of possible future therapeutic directions and mechanisms of action.

Control groups were classified into non-active, active, and non-comparative categories. Non-active controls included placebo. Active comparators consisted of standard-of-care pharmacological treatments. Single-arm non-comparative studies (e.g., open-label extensions and cohort studies) were incorporated to ensure a comprehensive assessment of long-term safety.

The primary efficacy outcome was the proportion of patients achieving a clinically meaningful response in endometriosis-associated pain (EAP), which included, but was not limited to, dysmenorrhea, NMPP, and dyspareunia. The response was defined as improvements exceeding established clinically important differences (CIDs) specific to the scales used in each study. Secondary efficacy outcomes included pain interference with daily activities and sleep (e.g., Brief Pain Inventory), health-related quality of life (QoL) (EHP-30) and changes in endometrioma size measured by ultrasound.

A safety profile was evaluated by the incidence of treatment-emergent adverse events (TEAEs), serious adverse events (SAEs), and discontinuation rates. Specific attention was paid to changes in bone mineral density (BMD) at the lumbar spine and femoral neck, venous thromboembolism (VTE) risk, and long-term risks of endometrial and breast cancer. Exploratory markers, including inflammatory biomarkers and angiogenic pathway parameters, were synthesized to provide insights into the drug’s mechanism of action.

We will exclude studies involving male participants, postmenopausal women, participants under 15 years of age, pregnant and breastfeeding women, participants with active malignancy (particularly hormone-dependent), current or history of thromboembolic disorders, uncontrolled or severe cardiovascular conditions, active liver failure or other significant liver diseases that may compromise drug metabolism.

We will exclude studies where the intervention is non-pharmacological, including surgical treatments (laparoscopy, hysterectomy, etc.), urogynecological physical therapy and other forms of physical or manual therapy, psychotherapy and sexological therapy, Complementary and Alternative Medicine (CAM), including herbal remedies, dietary supplements, essential oils, acupuncture, or homeopathy.

We will exclude studies where the comparator group involves the following: comparisons against non-pharmacological interventions such as surgical procedures, physical therapy, or alternative medicine; comparisons against unapproved or experimental agents that are not currently standard-of-care and whose efficacy is highly speculative;

We will exclude meta-analyses, systematic reviews, narrative reviews, scoping reviews, literature reviews, studies not published in English, conference abstracts, and expert opinions. The complete Systematic Review Protocol is provided in Supplementary Materials.

**Figure 1:**
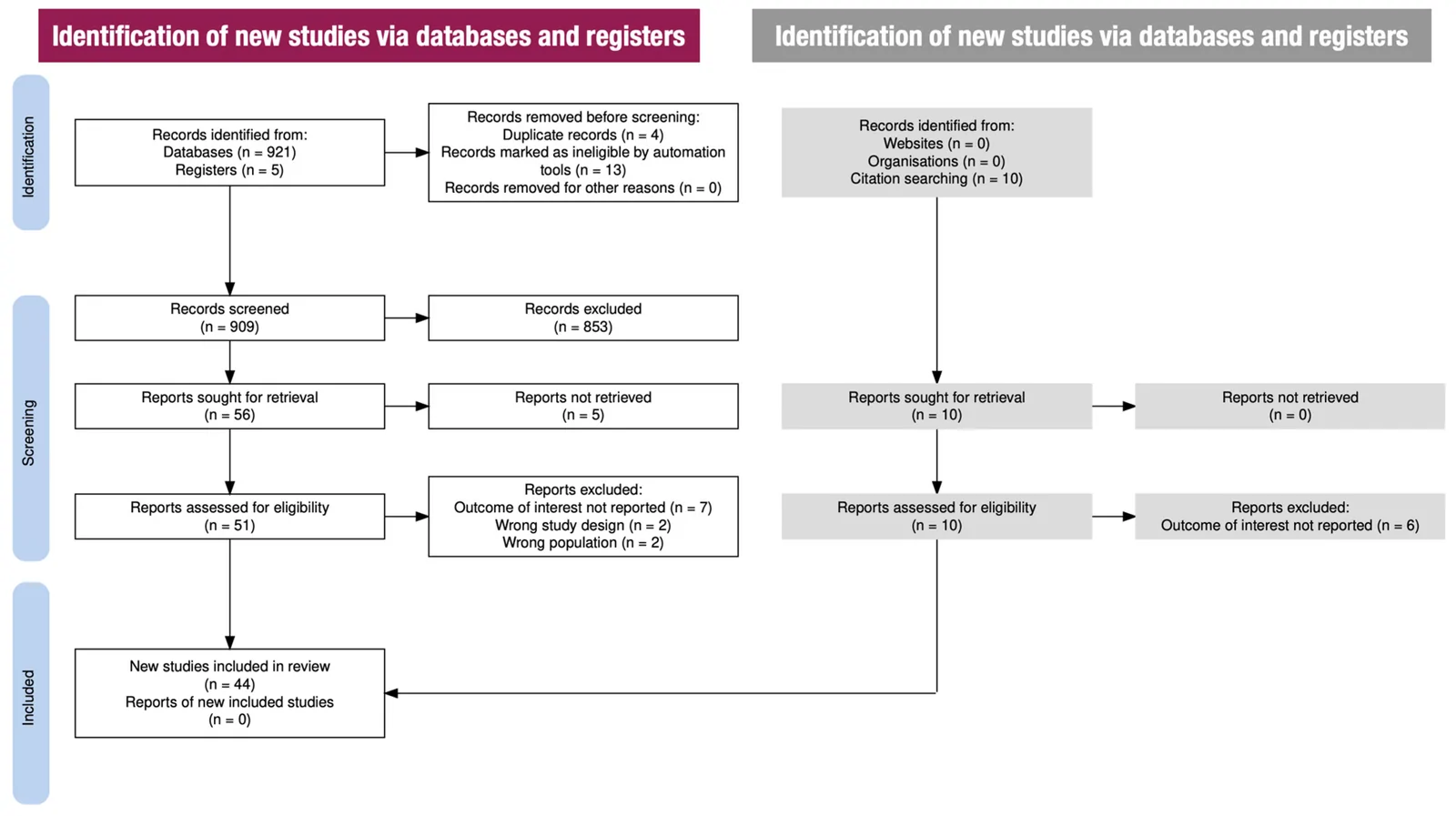
PRISMA 2020 flow diagram [7].

The search results were uploaded to Rayyan for de-duplication. Four reviewers independently screened titles and abstracts against the inclusion criteria, with disagreements resolved by consensus. The full texts of potentially eligible studies were assessed for final inclusion. The selection process is detailed in the PRISMA 2020 flow diagram (Figure 1).

Data were extracted independently by the same reviewers into an Excel-based form. The extraction focused on study characteristics, population demographics, intervention, control groups, and study funding sources. We also collected results for all primary efficacy, secondary, and safety outcomes. The results are presented in Table S1 Characteristics of the included studies, in the Supplementary Materials.

Risk of bias was assessed using the Cochrane Risk of Bias tool (RoB 2) for randomized controlled trials (RCT) and the ROBINS-I tool (Risk of Bias in Non-randomized Studies of Interventions) for non-randomized observational studies.

Due to the methodological and clinical heterogeneity of the included studies, a meta-analysis was not performed. Instead, a structured narrative synthesis was conducted. Results were grouped according to the specific drug. The certainty of evidence for each outcome was assessed using the Grading of Recommendations Assessment, Development, and Evaluation (GRADE). Details of the assessment are available in the Table S2 GRADE Summary of Findings.

## 3. Results

### 3.1. Pharmacotherapy in Endometriosis—Actual Recommendations

**Figure 2:**
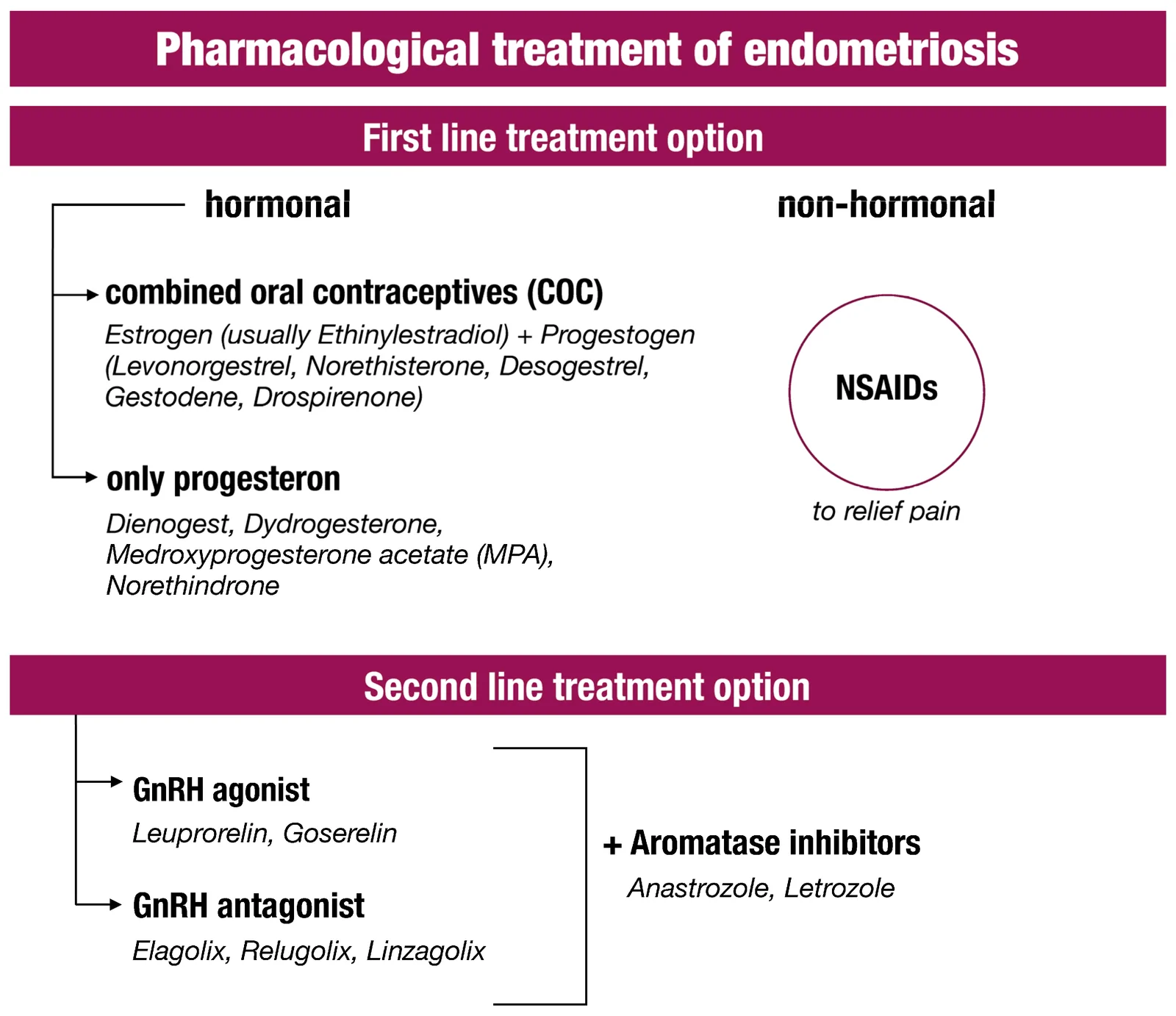
Pharmacological management recommendations for endometriosis [1,3].

According to the official recommendations of the European Society of Human Reproduction and Embryology (ESHRE) the patient can be offered hormonal treatment, analgesics or non-steroidal anti-inflammatory drugs (NSAIDs) to reduce EAP. Hormone treatment options include combined hormonal contraceptives, progestogens and as second-line treatment options: gonadotropin-releasing hormone (GnRH) agonist, GnRH antagonists and aromatase inhibitors. Aromatase inhibitors must be combined with one of the previously mentioned hormonal drugs. Hormonal treatment can be used in combination with analgesics or NSAIDs. Patient preferences, adverse effects, individual efficacy, cost and availability must be considered when choosing pain treatment options [1,3] (Figure 2).

### 3.2. 19-Nortestosterone Derivatives: Dienogest (DNG)

DNG is a drug that is recommended as a first-line treatment for any type of endometriosis. It is a 19-norprogestogen and progesterone derivative and it has a better tolerability profile compared to other progestogens, as well as a lower rate of side effects. It exhibits a strong progestogenic effect on the endometrium, in the absence of estrogenic and androgenic effects. In endometriosis, it is used for its anti-proliferative effect against the endometrium, and has an anti-inflammatory effect, inhibits ovulation and causes atrophy of the endometrium and endometriosis foci. DNG modulates gene expression in endometriosis lesions, particularly affecting pathways related to immune regulation, inflammation, cell signaling and adhesion [8]. DNG reduces estrogen secretion by affecting the hypothalamic–pituitary–ovarian axis.

In the treatment of endometriosis, it is concerned with reducing pain. There is no high-quality evidence confirming the superiority of DNG over combined oral contraceptives (COC) in reducing pain in patients with endometriosis; both are considered equally effective first-line options according to current research and guidelines. Studies have shown there is not superior therapeutic efficacy of DNG over COC—levonorgestrel 0.1 mg/ethinyl estradiol 0.02 mg [9,10]. The study by Yurtkal et al. is a prospective cohort study comparing DNG and two COC formulations (ethinylestradiol/DNG and estradiol valerate/DNG) in women with endometriosis. All groups showed significant reductions in pelvic pain—Visual Analogue Scale (VAS) scores and endometrioma size after treatment, but no statistically significant difference was found between the DNG and COC groups for pain reduction or cyst size. The authors concluded that cost-effective COCs are reasonable alternatives to DNG, given their similar efficacy and side effect profiles [10]. The conclusion of the study by Niakan G et al. reveals that both DNG and COC pills significantly reduce endometriosis-associated pain and improve QoL compared to placebo, but neither is superior to the other in terms of efficacy [9].

The study by Caruso et al. included 197 women diagnosed with endometriosis. In the study, the VAS was used to examine chronic pelvic pain and it was found that pain was reduced in both groups, with patients treated with DNF in one group and those in the other group treated with nomegestrol acetate plus 17 beta estradiol. Nevertheless QoL was higher in patients treated with DNG [11]. Despite all, DNG therapy is still recommended in the first-line setting by the World Endometriosis Society (WES).

That drug acts locally, which means that its activity focuses more on binding to receptors in the endometrium and endometriosis foci, and less on receptors in other tissues. This reduces the systemic effects of orally taken progestogens. One should be aware of the drug’s side effects, such as tender breasts, menstrual changes, weight gain. Bleeding usually occurs during the first 3 months of treatment [11]. A 2024 retrospective cohort study of 154 patients reported that the incidence of weight gain during the first 6 months of DNG therapy was 7.1% [12]. Additionally, a 2025 retrospective cohort study of 180 patients with endometrioma found that weight gain was more common in the short-term treatment group (<2 months), but did not provide a specific percentage [13].

Most patients do not discontinue therapy. The most common reasons why patients discontinue treatment are genital bleeding, decreased libido, and vaginal dryness. In a 2024 cohort study of 114 patients treated for up to 36 months, spotting, reduced sexual drive, vaginal dryness, and mood disorders were the main reasons for discontinuation, with spotting being the most common side effect. At 12 months, amenorrhea was reported in 77% of patients, increasing to 93% at 36 months. Discontinuation due to side effects occurred in 19% of patients, with 62% of these cases attributed to the above symptoms [14].

In summary, irregular bleeding, amenorrhea, decreased libido, vaginal dryness, and mood changes (including depression) are the most frequent side effects of DNG in endometriosis, with irregular bleeding and amenorrhea affecting up to 70–93% of patients in long-term studies, and depressive symptoms occurring in up to 36.7% [11,14,15].

Hormone therapy is not neutral to bone tissue. A study by Kim, S.E., Lim et al. which involved 44 women after endometriosis surgery, found that after 3 years of taking orally DNG 2 mg daily, there was a decrease in BMD at the lumbar spine of 4.4%, while in the femoral neck the decrease was 3.6%. The greatest decrease was after one year of therapy. It is also worth mentioning a study conducted by Park et al. where it was found that long-term therapy with DNG did not significantly reduce BMD [16]. However, it is important to note that DNG still has a smaller effect on BMD than other hormonal drugs. Longer-term studies of DNG (up to 3 years) show a gradual BMD decline, predominantly in the first year (2–2.7% at the lumbar spine), but this loss is still less pronounced than with GnRH agonists and is not associated with increased bone turnover markers [17].

There are no studies that confirm a higher risk of breast cancer associated with the use of DNG for the treatment of endometriosis. The most recent and robust evidence comes from a cohort study by Jhang K. et al. who found that DNG use for at least 6 months was not associated with an increased risk of breast cancer compared to GnRH agonists (adjusted hazard ratio [aHR] 1.01, 95% CI 0.75–1.37) [1]. Furthermore, short-term use (0.5–1.5 years) of DNG was associated with a reduced risk of breast cancer (aHR 0.72, 95% CI 0.53–0.99), while longer durations showed inconsistent associations [18].

DNG therapy is contraindicated in cases of pregnancy, lactation, severe liver disease, and hormone-dependent cancers such as breast cancer. It is also not recommended in pulmonary embolism and deep vein thrombosis because it raises the risk of thromboembolic incidents, but to a lesser extent than classic COCs. For example, a study by Bauerfeind et al. compared the risk of VTE between users of COCs containing estradiol valerate–DNG and those containing ethinyl estradiol–levonorgestrel [19]. This analysis included 11,616 estradiol valerate–DNG users and 18,681 ethinyl estradiol–levonorgestrel users, with a total of 17,932 and 29,140 women-years of observation, respectively. After adjusting for baseline differences using propensity score subclassification, the study found a significantly decreased VTE risk in estradiol valerate–DNG COC users compared to ethinyl estradiol–levonorgestrel COC users (hazard ratio 0.46, 95% CI 0.22–0.98). This analysis provides real-world evidence that estradiol valerate– DNG COCs are associated with a lower risk of VTE than the commonly used ethinyl estradiol–levonorgestrel COCs, supporting the safety profile of natural estrogen-based COCs in terms of thromboembolic risk [19].

The effect of DNG on fertility and its use in in vitro procedures (IVF) is being studied. In a study by Barra et al., higher pregnancy and live birth rates were observed in women with endometriosis undergoing an IVF preceded by 3 months of DNG therapy than in women who did not receive this treatment. The study involved 151 women, of whom 63 received DNG therapy before IVF and 88 did not. Implantation and pregnancy rates in women treated with DNG were 39.7% and 33.3%, respectively. In contrast, the results in women without DNG therapy were significantly lower. The implantation rate was 23.9% while the pregnancy rate was 18.2% [20]. In another study, written by Tamura et al., the results were not as satisfactory, and an even lower pregnancy rate was observed in women after IVF preceded by DNG therapy (6 women out of 33, 18.2%) than in a control group of women without this treatment (11 women out of 35, 31.4%) [21].

### 3.3. GnRH Antagonist: Relugolix and Elagolix

Relugolix and elagolix are non-peptide GnRH antagonists. Compared to analogs, they do not cause an increase in follicle-stimulating hormone (FSH), luteinizing hormone (LH) and intensification of symptoms in the initial period of treatment. In 2018, the Food and Drug Administration (FDA) approved elagolix 150 mg once daily or 200 mg twice daily for the management of moderate to severe EAP in premenopausal women, with a treatment duration of up to 24 months for the 150 mg dose or up to 6 months for the 200 mg dose [22]. In 2022 the FDA approved relugolix 40 mg, estradiol 1 mg and norethindrone acetate 0.5 mg (*Myfembree*, Myovant Sciences, Inc., Brisbane, CA, USA and Pfizer Inc., New York, NY, USA) as a once-daily oral combination therapy (CT) with a treatment duration of up to 24 months [23].

#### 3.3.1. Relugolix

SPIRIT 1 (NCT03204318) and 2 (NCT03204331) are two replicate, double-blind, multicenter, randomized, placebo-controlled study. Premenopausal women with a diagnosis of endometriosis or EAP were included in the study. A total of 638 women with endometriosis were qualified into SPIRIT 1 study and 623 into SPIRIT 2 study. In both studies patients were assigned equally between three groups: to the relugolix CT group (relugolix 40 mg, estradiol 1 mg, norethisterone acetate 0.5 mg), to the delayed relugolix CT group (relugolix 40 mg monotherapy followed by relugolix CT, each for 12 weeks) and to the placebo group. Patients were treated for 24 weeks [24] (Table 2).

Relugolix CT was observed to be similarly effective to delayed relugolix CT in the treatment of dysmenorrhea. Pain reduction on a Numerical Rating Scale (0 = no pain; 10 = pain as bad as you can imagine) (NRS) by at least 50% in the last 35 days of treatment without using more analgesics was declared by 75% of patients from the relugolix CT group, 72% from the delayed relugolix CT group and only 29% from the placebo group. Relugolix is not as effective in relieving NMPP. In this case, 62% patients from the relugolix CT group, 55% from the delayed relugolix CT group and 41% from placebo group responded to treatment [24].

The efficacy of long-term treatment with relugolix CT was assessed in SPIRIT LTE open-label extension study (NCT03654274). Of the 1261 patients from SPIRIT 1 and 2802 patients enrolled in the long-term extension study, 681 women received treatment (relugolix 40 mg, estradiol 1 mg, norethisterone acetate 0.5 mg) for 52 weeks and 502 patients received treatment for 104 weeks. The study observed that the relugolix CT had the same or better effectiveness at 52 and 104 weeks of treatment. The effectiveness increased most in patients who previously received placebo (75.6% of patients met the dysmenorrhea responder definition at week 52 and 80.4% at week 104). The highest effectiveness was maintained in patients who received the relugolix CT from the beginning (84.8% dysmenorrhea at 52 and 104 weeks, NMPP—73.6% at week 52 and 75.8% at week 104). A steady decrease in the intensity of NMPP was observed (reductions from baseline of 69.4% at 52 week and 58.6% at 104 week). This study also showed a decrease in analgesics use with duration of relugolix CT treatment [25].

In another randomized, double-blind study (NCT01458301) that investigated whether the effect of relugolix was dose-dependent, 487 patients with a diagnosis of endometriosis were assigned to groups: 99 to placebo group, 103 to relugolix 10 mg group, 100 to the relugolix 20 mg group, 103 to the relugolix 40 mg group and 82 to the leuprolin 3.75 mg group. Patients received treatment for 12 weeks. Pain intensity was measured using the VAS. A dose-dependent effect with the highest effectiveness for the 40 mg was observed in reducing pelvic pain (−10.4 from baseline) and dysmenorrhea pain. This effect was comparable to leuproline [26]. The study was extended to 24 weeks. At 24 weeks, the drug maintained its efficacy across the three doses mentioned, yet dose 40 mg still demonstrated the highest efficacy: −11.9 from baseline for pelvic pain, −29.5 for dysmenorrhea and −0.9 for dyspareunia. Relugolix demonstrated no efficacy in the treatment of dyspareunia at any dosage, even in this trial [24,27,28].

Relugolix treatment is not associated with the risk of SAEs, the most frequent being: headache, nasopharyngitis, hot flushes, metrorrhagia, menorrhagia, irregular menstruation, hyperhidrosis and BMD loss [24,26,27]. TEAEs occur more frequently the higher the dose of relugolix (comparison of doses 10 mg, 20 mg and 40 mg) [26]. The SPIRIT open-label study observed that the frequency of TEAEs did not increase over time nor did any new, previously unobserved TEAEs occur [25].

BMD loss increases with the relugolix dosage (comparison of doses 10 mg, 20 mg and 40 mg) and its duration of use (comparison of 12 and 24 weeks of treatment) [26,27]. After the 12 weeks of treatment the effect of relugolix 40 mg on BMD loss was consistent with the effect observed after leuprorelin administration [26]. However, after 24 weeks of treatment, BMD loss in the group receiving 40 mg of relugolix was −2.91%, and also increased in the other groups (from −1.88% to −2.34% for relugolix 10 mg; from −2.09% to −2.94% for relugolix 20 mg). The lowest BMD loss is observed in patients receiving relugolix CT: −0.74%, while BMD loss in patients receiving delayed relugolix CT is −2% [24].

The safety of long-term treatment with relugolix CT was assessed in SPIRIT LTE open-label study (NCT03654274). Up to 24 week of treatment, BMD loss was less than 1% in patients receiving relugolix CT and stabilized over time [24]. However, in patients who took delayed relugolix CT in the initial period of treatment, a tendency towards recovery of BMD loss was observed. BMD loss in these patients in week 24 was −1.86% of baseline, while in week 104 it was −0.56% of baseline [25].

The SPIRIT LTE study observed a return of menstruation in 90% of patients 2 months after completion of treatment of relugolix CT. Pregnancy, menopause, surgery, or other treatments prevented this in the remaining patients [25]. In another study (NCT01452685), after completing 24 weeks of treatment of relugolix, a return of menstruation was observed in 95% of patients. The time from the end of treatment to the return of menstruation was dose-dependent: the lower the dose, the shorter the time (21 days for relugolix 10 mg, 36.9 days for relugolix 40 mg). For patients receiving leuproline, this time was 73.3 days [27].

Contraindications to the use of relugolix are: pregnancy and breastfeeding, high risk of thrombotic or thromboembolic disorders, uncontrolled hypertension, hormone-dependent cancers, osteoporosis, drug hypersensitivity, liver failure, undiagnosed and abnormal vaginal bleeding [23].

#### 3.3.2. Elagolix

The efficacy of elagolix in the treatment of endometriosis was proven by Elaris EM-I and Elaris EM-II studies in 2017. These are two double-blind, randomized studies during which after 3 months a reduction in NMPP was observed in 52.4% of patients receiving elagolix 150 mg once daily or 200 mg twice daily. An amount of 200 mg is more effective in reducing dysmenorrhea (74.1% of patients from higher-dose group) than 150 mg dose (44.9% of patients from lower-dose group) [29].

In the other study (NCT03213457) investigating the efficacy and tolerability of the elagolix, 681 premenopausal women with endometriosis were assigned between three groups: 389 to group receiving elagolix 200 mg twice daily with hormonal add-back (AB) therapy (1 mg estradiol/0.5 mg norethindrone acetate once daily), 98 to group receiving elagolix 200 mg twice daily monotherapy for 6 months followed by elagolix with AB therapy and 194 to placebo group. Patients were treated for 12 months [28]. After 6 months of treatment 62.8% of patients from elagolix with AB therapy group experienced a decrease in dysmenorrhea by at least two points in NRS without using more analgesics and only 23.7% from the placebo group. At 12 months, the proportion of patients was 63.8%. NMPP reduction after 6 months achieved 51.3% of patients from this group compared to 36.8% of patients from the placebo group. Following the 12-month blinded period, the trial transitioned into an open-label extension phase where all patients received elagolix 200 mg with AB therapy for an additional 36 months, resulting in a total treatment duration of up to 48 months. This effectiveness was maintained at 36 months of treatment [30]. Elagolix 150 mg once-daily monotherapy is ineffective in treating dyspareunia compared with placebo, but improvement was observed in 44.1% of patients receiving elagolix 200 mg twice daily monotherapy [29]. In the group receiving Elagolix 200 mg with AB therapy, improvement in dyspareunia was achieved by 45.8% of patients at 6 months, compared with 30.7% in the placebo group [28].

Most patients taking elagolix experienced mild adverse effects: the most common are hot flushes, headache, nausea, BMD loss, higher levels of serum lipids [22,25]. BDM loss of elagolix is also dose-dependent, reaching significantly higher values than in the case of relugolix. After 6 months of treatment, 20.9% of patients receiving 200 mg of elagolix twice daily experienced a BMD loss greater than 5% [29]. In another study, patients receiving elagolix 200 mg with AB therapy or placebo had a BMD loss of ≤1%, whereas patients receiving elagolix 200 mg monotherapy had a significantly greater BMD loss after 6 months of treatment: −2.88% from baseline in the lumbar spine. After the addition of AB therapy, BMD loss stabilized but no recovery trend was observed. In the 12th month of treatment, the loss remained at a level similar to that observed in the 6th month. After 36 months of treatment BMD change was relatively stable at the total hip and lumbar spine, but at the femoral neck it was −1.39% from the baseline [30].

### 3.4. New Perspectives

Current treatments for endometriosis are focused on alleviating symptoms experienced by patients and slowing disease progression. Recently, numerous new therapeutic approaches have emerged, targeting the complex pathophysiological mechanisms underlying the development of the disease. In some patients, the disease still cannot be completely cured, which is why research into new medications remains extremely important.

#### 3.4.1. Drospirenone (DRSP)

DRSP, a synthetic progestogen with weak anti-androgenic activity, has emerged as a promising compound in the treatment of endometriosis. In combination with estrogens, it is a component of monophasic oral contraceptives [31]. A recent study conducted in Japan suggests that therapy combining estetrol (E4) with DRSP may contribute to reducing EAP. The study included 162 women diagnosed with endometriosis, who were randomly assigned to two groups. The first group received a combination of 15 mg E4 and 3 mg DRSP daily for 24 days, followed by 4 days of placebo, repeated for six cycles. The second group received a placebo throughout the study. The results showed that 36.4% of patients in the treatment group reported a ≥50% reduction in pain intensity, compared to 12.3% in the placebo group. The treatment also decreased both the number and size of endometrial cysts. The volume of the largest ovarian cyst in the treated group decreased by approximately 44.5% compared to placebo [32]. Other centers are currently conducting studies to further evaluate the effect of the DRSP/E4 combination on cyst size [33].

Therapy with DRSP carries a lower risk of thrombosis than traditional estrogen–progestogen therapies, with the combination of E4/DRSP showing more favorable effects than ethinylestradiol (EE/DRSP). This is supported by differences in coagulation and fibrinolysis parameters measured in a group of 82 women. D-dimer levels were 4.7 times higher in the EE/DRSP group, while protein S and TFPI (Tissue Factor Pathway Inhibitor) levels were lower compared to the E4/DRSP group. The activated protein C sensitivity ratio (APC-SR), used to assess the risk of VTE, increased fourfold in the EE/DRSP group compared to the E4/DRSP group [34]. Additionally, the E4/DRSP combination has a smaller effect on patients’ triglyceride levels (in patients treated with E4/DRSP triglycerides increased by 24%, whereas in patients taking EE/DRSP they increased by as much as 65.5%). Moreover, it does not significantly affect blood glucose levels, which makes it particularly advantageous for patients whose endometriosis coexists with metabolic diseases [35].

#### 3.4.2. Astaxanthin (AST)

Endometrial cells developing outside the uterus lead to chronic inflammation, pain, and difficulties with conceiving. Among the emerging new pharmacological treatment options, AST stands out as a carotenoid with strong antioxidant properties [36]. In patients with endometriosis, decreased activity of antioxidant enzymes and elevated levels of reactive oxygen species (ROS) activate the NF-κB pathway, leading to the production of pro-inflammatory cytokines in peritoneal fluid, serum, and follicular fluid, negatively impacting fertility.

In 2023, a study was conducted with 50 women aged 20–40 years suffering from endometriosis-associated infertility. Participants were divided into two groups: one received AST, and the other received a placebo. The study aimed to assess the effects of the carotenoid on inflammation, oxidative stress, and fertility. Data analysis showed that women receiving AST experienced reductions in pro-inflammatory cytokines—interleukin-1B (IL-1B) and Tumor Necrosis Factor α (TNF-α) in serum, and interleukin 6 (IL-6) and TNF-α in follicular fluid. Additionally, markers of oxidative stress decreased, suggesting a potential positive effect on fertility. Women treated with AST also had a higher number of collected and mature oocytes, supporting its potential reproductive benefits [37].

In another study with 51 women, the anti-inflammatory effect of AST was confirmed, showing significant decreases in IL-6, IL-8, and vascular endothelial growth factor (VEGF) levels in both serum and follicular fluid after therapy. These results further support the beneficial effect of AST on fertility, although patients with endometrial lesions were excluded from this study [38]. The effectiveness in reducing inflammation was also observed in patients with polycystic ovary syndrome (PCOS). It was found that AST supplementation lowers levels of TNF-α, IL-18, and IL-6, and that taking it for at least 3 months or at doses above 12 mg/day may reduce the level of the systemic inflammatory marker C-reactive protein (CRP) [39].

The significance of AST in the treatment of endometriosis is growing, as there is increasing discussion regarding its influence on steroid metabolism, including hormones such as estrogens, which are a non-negligible risk factor for endometriosis [40]. However, these findings remain preliminary.

#### 3.4.3. D2 Receptor Agonists

The development of endometrial lesions outside the uterine cavity requires continuous formation of new blood vessels. In this process, VEGF plays a crucial role. For this reason, inhibiting angiogenesis through the use of dopamine D2 receptor agonists has become one of the methods employed in endometriosis therapy [41]. The use of D2 receptor agonists is justified due to the reduced expression of these receptors in ectopic endometrial cells and the increased expression of VEGF [42].

In a pilot study ”Nonhormonal therapy for endometriosis: a randomized, placebo-controlled, pilot study of cabergoline versus norethindrone acetate” with nine patients, the efficiency of a cabergoline was compared with a synthetic progestogen (norethisterone acetate). Patients were randomly assigned to two groups: the first received 5 mg of progestogen daily with placebo twice weekly, while the second received 0.5 mg cabergoline twice weekly plus daily placebo for 6 months. The average pain intensity (assessed by the VAS) decreased by 83% in the cabergoline group and 57% in the progestogen group. Additionally, patients receiving the cabergoline showed decreased levels of VEGF receptor 1 (VEGF-R1), whereas levels increased in the progestogen group. Cabergoline is also considered relatively safe and does not interfere with pregnancy progression. However, the small number of patients is a limitation of this study [43]. Animal studies have also demonstrated that D2 receptor agonists may inhibit angiogenesis similarly to current anti-VEGF drugs, reducing vessel density while increasing the proportion of mature vessels and apoptotic cells. In the D2 agonist group, vessel density was reduced by ~29.7%, compared to ~34.3% in the anti-VEGF group. The effectiveness of angiogenesis inhibition depends on the maturation stage of the vessels at the time of therapy [44]. It has been shown that the use of cabergoline, in addition to its antiangiogenic effect, may also protect against endometriosis through another mechanism—protection against oxidative stress by inhibiting the TRPM2 channel in neutrophils [45].

Currently, the estetrol/drospirenone (E4/DRSP) combination is supported by the most robust clinical data, demonstrating efficacy and a favorable safety profile in larger randomized trials. In contrast, evidence for astaxanthin and dopamine D2 receptor agonists remains preliminary. While astaxanthin shows promising anti-inflammatory and fertility-enhancing effects in small clinical studies, data for D2 agonists (e.g., cabergoline) are largely restricted to animal models and very small pilot trials. Despite limited evidence, these emerging therapies attract clinical interest because they do not suppress ovulation—they may serve as an alternative for patients planning a pregnancy.

### 3.5. Endometriosis, Immunology and Targeted Therapy—Is There a Chance for a Breakthrough in Treatment?

Endometriosis is a disease characterized by chronic, ongoing inflammation, which may result from imbalance between individual elements of the immune system. Macrophages, natural killer (NK) cells, neutrophils and the mediators they release deserve special attention, as they may be the targets for drugs that have not been used in the treatment of endometriosis yet.

The cell populations secrete cytokines and growth factors that facilitate angiogenesis, cellular proliferation, and the invasive potential of endometriotic lesions. Among these mediators are IL-1, IL-6, and IL-8, as well as VEGF. Moreover, these cells display dysregulated cytokine expression, as demonstrated by altered concentrations in the peritoneal fluid of affected individuals when compared with healthy controls.

VEGF is implicated in the pathogenesis of endometriosis by stimulating neovascularization within endometriotic lesions and is found at elevated concentrations in the peritoneal fluid of affected patients. Consequently, particular attention should be directed toward its biological activity, as this pathway may represent a promising target for the development of novel therapeutic strategies [46,47,48]. To date, a single case report published in 2021 has described the use of the anti-VEGF monoclonal antibody bevacizumab in a 46-year-old woman with endometriosis, in whom standard hormonal therapy had proven ineffective in alleviating severe dysmenorrhea. Following confirmation of the diagnosis, the patient received three intravenous doses of bevacizumab administered at 2 weeks intervals. Upon completion of this regimen, the pain symptoms resolved completely, while positron emission tomography (PET) demonstrated reduced metabolic activity of the lesions compared with the pre-treatment findings. Moreover, 1 month after administration of the final dose, resection of additional lesions was performed. Histopathological examination revealed an increased expression of estrogen and progesterone receptors within the lesions, which enhanced sensitivity to conventional hormonal therapy. It is noteworthy, however, that despite these benefits, increased fibrosis within the lesions was also observed [49].

Elevated concentrations of proinflammatory cytokines, such as IL-6, in the peritoneal fluid of affected women suggest that these proteins may represent a potential target for immunotherapy in endometriosis. The action of IL-6 involves inhibition of apoptosis in endometrial cells within lesions, as well as enhancement of their invasive potential. Additionally, IL-6 exerts a range of effects on immune cells, promoting inflammatory activity. The anti-IL-6 monoclonal antibody tocilizumab, previously used in the treatment of chronic inflammatory joint diseases, has recently attracted interest as a potential therapy for endometriosis. In 2020, results from a study using an animal model of the disease were published: in rats from the experimental group (*n* = 14), lesion volume was reduced by an average of 50 mm^3^, and the density of IL-6 receptors within the lesions decreased following a 4-week course of tocilizumab compared with the control group (*n* = 5). Histopathological evaluation based on the intensity of immunohistochemical staining revealed changes in epithelial thickness, with over 40% of cases showing reduced staining, indicative of thinning of the ectopic endometrial epithelium, without affecting normal endometrium in the uterus [50,51,52].

The next element in the pathogenesis of endometriosis is the IL-1 family, which includes both pro-inflammatory and anti-inflammatory mediators. In addition to IL-1 itself, this group comprises, among others, IL-36, IL-37, and IL-38. The IL-1 family plays a particularly important role in regulating the inflammatory response due to its influence on the majority of immune system cells. In 2024, a study was conducted to evaluate the effect of an IL-1 receptor antagonist (Anakinra) on the course of endometriosis. The study involved women with confirmed endometriosis who primarily experienced moderate to severe dysmenorrhea. During three consecutive menstrual cycles, participants received a daily dose of 100 mg Anakinra. The results were compared with three menstrual cycles during which the same participants received a placebo. Pain intensity was assessed using a graded scale (0–3), and QoL was evaluated with a standardized questionnaire. Although the improvement in dysmenorrhea was modest, a statistically significant improvement in QoL was observed among patients who completed all treatment cycles (*n* = 15). An important aspect of this therapy is the fact that Anakinra likely does not affect fertility—the participants reported no changes in cycle length and continued to menstruate, which suggests preserved ovulation. Nevertheless, these findings should be interpreted with caution, given the limited sample sizes and the preliminary nature of the available evidence [53,54,55].

Another potential target for immunotherapy may be the immune checkpoint associated with the CD47 protein, which is directly linked to the function of macrophages—cells that play a crucial role in the development and progression of endometriosis. This protein participates in a signaling pathway that ultimately leads to the inhibition of macrophage-mediated phagocytosis [56]. As a result, peritoneal macrophages, despite their increased activity, are unable to eliminate erythrocytes, cellular debris, and damaged tissue fragments. Based on this mechanism, a study was conducted and published in 2022. Tissue samples from endometriotic lesions were collected from patients with histologically confirmed endometriosis during laparoscopy. Stromal cells were then isolated from these samples and cultured. In parallel, primary endometrial stromal cells were isolated from healthy mice. Immunofluorescence and flow cytometry analyses revealed elevated CD47 expression in cells derived from endometriotic lesions, which enabled them to evade phagocytosis. To verify the practical applicability of these findings, the CD47 gene was inactivated in endometrial stromal cells. Using both in vivo and in vitro macrophage co-culture models, researchers demonstrated increased susceptibility of stromal cells to phagocytosis as well as apoptosis. Comparable results were also achieved through the use of anti-CD47 antibodies [57,58].

Immunotherapy for endometriosis remains a developing field. Ongoing clinical trials are currently assessing the efficacy and safety of the monoclonal antibody HMI-115, which targets and blocks the prolactin receptor, as a potential treatment for the disease. So far, information has been released regarding the progress of the phase II clinical trial. The study involved 142 women aged 18 to 49 years with moderate to severe endometriosis diagnosed within the past 10 years. Participants received the antibody for a duration of 12 weeks, after which pain intensity was compared with that of a placebo group. The preliminary results reported by the investigators are promising, indicating a statistically significant reduction in pain. However, further research is ongoing in subsequent trial phases, and the currently available data remain limited [59].

When it comes to immunotherapeutic approaches, emerging therapeutic strategies may appear promising; however, the current evidence remains preliminary, particularly due to the small study populations and the presence of single case reports. Furthermore, findings derived from animal models require further investigation before these approaches can be translated into clinical treatment for patients with endometriosis. These limitations, together with the risk of bias and the certainty of the evidence assessed using the GRADE framework, should be carefully considered when evaluating and pursuing novel therapeutic options for endometriosis.

## 4. Discussion

### 4.1. Comparison with Current Guidelines (ESHRE/ASRM)

This review confirms the well-established position of DNG within the guidelines, given its high efficacy in reducing pelvic pain and preventing postoperative recurrences of endometriomas [8,60]. At the same time the analyzed studies demonstrate the high efficacy of oral GnRH antagonists (relugolix and elagolix) [24,25,28,29,30]. These medications are valuable second-line therapy, providing rapid pain relief, while the concurrent use of add-back therapy prevents bone loss [24,25].

However, it should be noted that the current ASRM and ESHRE guidelines focus primarily on symptom management and ovarian suppression, which often fails to address the underlying inflammatory pathophysiology of the disease and is unsuitable for patients actively trying to conceive [3,37]. The emerging biologics and immunotherapies discussed in this review (e.g., tocilizumab, anakinra) have not yet been included into clinical guidelines. This is fully justified, as the premature implementation of these therapies would pose risks to patients, and their potential clinical application requires prior verification.

### 4.2. Clinical Implications

The choice of endometriosis pharmacotherapy must be highly individualized, carefully balancing the patient’s reproductive plans, comorbidities, and the risk of TEAEs [3,12]. Although DNG is highly effective and generally well-tolerated, its long-term use requires monitoring due to side effects such as irregular bleeding, mood swings, and a potential impact on BMD [14,15,17]. This review confirms that DNG is applicable across various disease phenotypes, including deep infiltrating endometriosis and endometrial cysts. Its favorable safety profile and efficacy across diverse endometriosis phenotypes position it as a basis of modern care for patients with this disease. Despite all advantages, because data on the long-term risk of malignancy, including breast cancer, is limited, ongoing patient monitoring is advised. A study conducted by researchers in Taiwan showed a negligible effect of DNG therapy on increasing the risk of breast cancer in patients. The analysis included 1.070 women, of whom 19 were diagnosed with a BI-RADS 4 lesion. After biopsy in 18 patients, breast cancer was confirmed in one. Based on these data, the percentage of patients who developed breast cancer is 0.093% [61,62]. Due to the limited research on the effect of DNG on the development of breast cancer, caution and regular testing in patients at risk are recommended.

Research on GnRH antagonists indicates their more significant role in the treatment of endometriosis in the future. Relugolix CT appears to be a more effective option than elagolix in reducing dysmenorrhea and NMPP. Furthermore, the percentage of patients who respond to relugolix CT treatment increases with the duration of use, which is not observed with elagolix [25,30]. Significant reduction in dysmenorrhea in patients taking the relugolix CT contributes to the decrease in the use of opioids compared to patients from placebo group, which reduces patients’ exposure to the AEs of analgesic therapy [24,25]. Unfortunately compared to elagolix 200 mg, relugolix CT is not effective in reducing dyspareunia compared to placebo [24,25]. This may be due to limitations in the subjective assessment of pain intensity or the mechanism of action of relugolix—GnRH antagonist, which inhibits ovulation, menstruation and the activity of endometriosis foci in the endometrium, while dyspareunia may have non-hormonal causes (including neuronal sensitization, muscular disorders, adhesions, fibrosis). Therefore, GnRH antagonist CT combined with urogynecological physiotherapy, sexological, or psychological consultations has the potential to significantly improve the QoL for patients suffering from endometriosis.

GnRH antagonist monotherapy is not suitable for long-term use due to its hypoestrogenic effects [22,23,25,30]. New opportunities in their application were made possible by the development of antagonist GnRH CT with estradiol and norethisterone acetate, which limits the side effects resulting from the drop in estrogen levels. The lower BMD loss suggests the possibility of its long-term use. Elagolix 200 mg monotherapy is FDA-approved for the treatment of endometriosis for up to 6 months [22]. However, elagolix 200 mg with AB therapy was used for 48 months with good efficacy and without progressive BMD loss [30]. Relugolix CT was used for 26 months, which is 2 months longer than FDA recommendations, maintaining high efficacy without an increase in AEs. The mean BMD loss at Week 104 of relugolix CT treatment was −0.56%. Additionally, unlike with elagolix, a tendency towards the recovery of BMD loss is observed after the inclusion of estradiol and norethisterone acetate in relugolix monotherapy. This argues for the greatest safety of relugolix CT among elagolix [25].

Relugolix CT may be better option than leuproline because it is taken orally, which is a more comfortable option for patients than injection. Compared to leuproline, relugolix causes a faster decline in estrogen, progesterone, LH and FSH—relugolix in the send week of treatment and leuproline in the fourth week, which translates into faster treatment results. So, relugolix shows a faster onset of action and a faster return of menstruation, which may be an advantage for patients planning a pregnancy. There is a need for continued follow-up of the patients who participated in the trials to track reported pregnancies. Both drugs exhibit a very high incidence of TEAEs among patients (95.1% of patients receiving relugolix 40 mg and 97.5% of patients receiving leuproline) [26].

Furthermore, both ORILISSA (elagolix) and MYFEMBREE (relugolix) are orally administered tablets with a dose-dependent effect, enhancing the ease and convenience of use. However, both therapies currently represent a significant financial burden for uninsured patients in the United States, while in Europe these drugs are not reimbursed for the treatment of endometriosis [22,23].

The next drugs that may be considered as add-on therapy for endometriosis are anastrozole and letrozole. They are third generation aromatase inhibitors, which inhibit the conversion of androgens into estrogens. As a result, anastrozole/letrozole lowers estrogens levels. They are used in endometriosis treatment and typically combined with progestins or oral contraceptives. Endometriosis lesions produce aromatase, which increases local concentration of estrogenes and stimulates the lesions to grow and intensify inflammation. By blocking aromatase, it helps to reduce size of the lesions, reduce inflammation and in result relieve pain. Anastrozol or Letrozole therapy is not a first-line treatment. They are both used off label. They are reserved for selected cases [63]. Common side effects of both include hot flashes, asthenia, arthritis, pain, arthralgia, hypertension, depression, nausea, rash, osteoporosis, and fractures, as the result of low estrogens.

### 4.3. Future Research Directions

One potential direction in the evolution of endometriosis treatment is the development of immunotherapeutic and targeted non-hormonal therapies, which remain at an early stage of investigation. The evidence available to date only provides a novel perspective on this field—its interpretation is limited by the low certainty of evidence and high risk of bias.

Because altered cytokine concentrations in peritoneal fluid and impaired macrophage function drive disease progression, future research must prioritize immunomodulators [46,64]. Preliminary data on interleukin inhibitors (anti-IL-6, anti-IL-1) and CD47 immune checkpoint blockade provide a basis for novel disease-modifying strategies [51,53,57,58].

The use of anti-CD47 antibodies has resulted in increased susceptibility of endometrial stromal cells to phagocytosis and apoptosis [46,47]. Targeted therapy with bevacizumab also represents a novel therapeutic approach to endometriosis. A case report described a woman in whom treatment with this drug led to a reduction in pain symptoms and metabolic activity of endometriotic lesions [49]. Therefore, bevacizumab therapy requires further evaluation and careful monitoring of its long-term effects. In line with the growing interest in this topic, information is emerging regarding ongoing clinical trials. One such study is investigating the efficacy of the HMI-115 antibody in alleviating pain in patients with endometriosis [49]. Further research in this direction is essential to clarify the underlying mechanisms and identify new therapeutic possibilities for endometriosis within the fields of immunology and targeted therapy.

Inhibiting the formation of new blood vessels limits the development and growth of endometrial lesions; hence, targeting VEGF has become a goal of new therapeutic approaches. The use of dopamine D2 receptor agonists—such as cabergoline—may not only suppress the growth of endometrial lesions, but also reduce the pain experienced by patients [43,44]. D2 receptor agonists may be as effective in relieving pelvic pain in endometriosis patients as conventional hormonal therapy. Similar effects were achieved using anti-VEGF monoclonal antibody—bevacizumab. Innovative anti-angiogenic therapies thus represent a promising option for endometriosis treatment. Therapy combining dopamine receptor agonists with immunotherapy demonstrated regression of pathological endometrial tissue, reduced histological scores, and stimulated a beneficial immune response, specifically an increase in CD25+ and CD5+ cells [65]. This provides evidence that combining various forms of non-hormonal endometriosis therapy is advantageous. It results in a synergistic positive effect, leading to better disease treatment outcomes.

AST, known for its potent antioxidant properties, may be an excellent therapeutic option for chronic inflammatory conditions, such as endometriosis. AST, like the previously mentioned biologics—tocilizumab and anakinra—may prove to be an excellent therapeutic option, particularly for patients planning to conceive [37]. Furthermore, for patients undergoing infertility treatment, non-hormonal substances such as AST and dopamine D2 receptor agonists (cabergoline) hold promising clinical implications, as they appear to inhibit angiogenesis without blocking ovulation [37,43,44].

Modern, non-hormonal treatment for endometriosis helps avoid complications such as thrombotic events, mood swings, and weight gain. A promising compound currently under investigation is dichloroacetate (DCA) (a glycolysis inhibitor), which restores normal metabolism and reduces the elevated levels of pro-inflammatory lactates in patients [66]. To date, studies on mouse models of induced endometriosis have demonstrated a reduction in endometrial lesions following DCA treatment (*p* = 0.02), as well as a decrease in lactate levels (*p* = 0.0360) [67].

Conducting multicenter RCTs with adequate statistical power is essential to definitively determine the long-term safety, optimal dosing, and the impact of these therapies on human fertility. Concurrently, broader epidemiological studies are required to clearly establish the long-term effect of DNG on breast cancer risk and bone metabolism, which remains a subject requiring further clarification [17,61].

## 5. Strengths and Limitations

One of the key strengths of the present review is the applied methodological approach—we strictly followed the PRISMA 2020 guidelines and registered the protocol in the PROSPERO database. This ensured transparency and narrowed down reporting bias. The screening process performed by several reviewers was versatile and covered 921 records. It minimized researcher bias and human error. To evaluate the validity and strength of included evidence we used the Cochrane Risk of Bias as a quality assessment tool. Additionally, we declare that there is no conflicts of interest regarding the publication of this article. Furthermore, this review synthesizes multiple and novel treatment approaches—both standard hormonal treatment as well as methods different from the actual pharmacotherapy recommendations. By including these aspects, we provide an extensive vision for researchers and specialists to seek and explore various treatment methods.

Despite its strengths, this review has several limitations. The first aspect to consider is the exclusion of subscription-based commercial databases, specifically Embase and Scopus. Due to institutional subscription constraints and the non-funded nature of this academic initiative, our primary electronic search relied on MEDLINE. This might result in the exclusion of some valuable data published in a different literature, although Medline covers a significant portion of the international biomedical literature. While we attempted to maximize search sensitivity by integrating Google Scholar, searching trial registries (ClinicalTrials.gov), and conducting manual backward citation searches of eligible records, the reliance on a single primary database introduces a potential risk of database bias and missing eligible studies.

The results of this systematic review should be interpreted in the context of the varying quality of the underlying evidence. Significant methodological and clinical heterogeneity among the included studies precluded a formal meta-analysis. While data on DNG and GnRH antagonists typically come from robust, large-scale phase 3 RCTs (e.g., the SPIRIT program for relugolix) [24,28], the certainty of evidence for emerging targeted therapies is often low or very low [42,43,53]. A significant proportion of reports on monoclonal antibodies and angiogenesis inhibitors are based on early-phase exploratory studies, in vitro models, or animal studies (e.g., the rat model for tocilizumab) [51]. Moreover, several pilot clinical trials testing novel substances were characterized by serious imprecision due to extremely small study groups (e.g., only nine patients in the cabergoline study and 15 patients who completed full cycles in the anakinra study) [43,53]. Finally, relying on observational cohort studies to assess long-term safety profiles carries an inherent risk of confounding bias and selection bias, highlighting the need for more rigorous analyses based on long-term follow-up [14,18].

## 6. Conclusions and Summary

GnRH antagonists are drugs with great potential in the pharmacotherapy of endometriosis; they demonstrate high efficacy and, when used in CT, safety for long-term use. However, they are currently difficult to access for most patients, unlike DNG which shows high efficacy but requires further research on its long-term safety (Table 3).

Currently recommended treatments do not affect the factors causing the development of the endometriosis [1,3]. The search for endometriosis treatment options using immunotherapy offers new hope. The rapid advancement of immunology encourages the search for innovative therapeutic approaches within this field. Consequently, a deeper understanding of the key mechanisms underlying the functioning of immune cells may serve as a foundation for the development of novel and promising treatment strategies for endometriosis. This outlines a potential new direction in endometriosis care: exploring a shift from routine hormonal suppression toward precision, disease-modifying drugs. However, these approaches are still in their early stages and strictly require rigorous validation in clinical trials. Studies of peritoneal fluid from women with clinically confirmed endometriosis have demonstrated elevated concentrations of IL-1 family interleukins compared with healthy controls [64]. Once again, such findings open the way for the exploration of new therapeutic strategies. The main advantage of such therapies would be their direct action on one of the underlying causes of the disease—chronic inflammation [39].

Endometriosis is one of the most common causes of female infertility. Chronic inflammation affects the quality of oocytes and sperm and interferes with embryo implantation. Therefore, drugs that influence the concentration of cytokines, prostaglandins, and free radicals may prove beneficial for patients planning pregnancy.

It is essential to clearly distinguish established, clinically approved treatments (such as dienogest and GnRH antagonists) from experimental therapies (such as biologics or targeted immunotherapies), which still hold a strictly investigational status. This highlights a key paradigm shift in endometriosis care: the transition from routine hormonal suppression to precision, disease-modifying drugs that still require rigorous validation in clinical trials.

## Figures and Tables

**Table 1:** The research question.

Population	Intervention	Comparison	Outcome
Premenopausal women aged 15–50 years with diagnosis of endometriosis	Pharmacological interventions: Progestins GnRH Antagonists GnRH Agonists Drug combinations Immunotherapy agents	Placebo No treatment control groups Active control groups with any current standard pharmacological treatment for endometriosis	Endometriosis-associated pain Quality of life Endometriosis lesion size Comprehensive safety profile Exploratory markers

**Table 2:** Comparison of Spirit 1 and 2 results [24].

	SPIRIT 1	SPIRIT 2
Number of participants	638	623
Patients whose endometriosis was diagnosed surgically	434 (68%)	415 (67%)
Patients taking opioids for pain relief at baseline	185 (29%)	288 (47%)
Patients responding to treatment for dysmenorrhea in the relugolix CT group	158 (75%)	155 (75%)
Patients responding to treatment for NMPP in the relugolix CT group	124 (58%)	136 (66%)
The incidence of adverse events in the relugolix CT group	71%	81%
Mean change in lumbar spine BMD	–0.70%	–0.78%
Patients terminated study participation early	98 (15%)	115 (18%)

**Table 3:** Dosage of the described drugs.

Drug	Dose
Diemono, Visanne	Dienogest 2 mg orally once daily
Elagolix	150 mg once daily or 200 mg twice daily
Myfembree	Relugolix 40 mg, estradiol 1 mg and norethindrone acetate 0.5 mg orally once daily
Anastrozole (add-on therapy)	1 mg orally once daily
Letrozole (add-on therapy)	2.5 mg orally once daily
Estetrol(E4) + Drospirenone (DRSP)	Estetrol (E4) 15 mg + drospirenone (DRSP) 3 mg taken for 24 days followed by 4 days of placebo per cycle, for a total of 6 cycles (24 weeks) [64]
Cabergoline	0.5 mg twice weekly
Anakinra	100 mg daily for three menstrual cycles
Astaxanthin (AST)	6 mg once daily

## Data Availability

All data analyzed during this systematic review are included in this published article. The primary data sources were obtained from the original published studies cited in the references.

## Supplementary Materials

The following supporting information can be downloaded at: https://www.mdpi.com/article/10.3390/jcm15187238/s1, Table S1: Characteristics of the included studies; Table S2: GRADE Summary of Findings, Systematic Review Protocol, Systematic Review Search Strategy. Suppmentary Materials File S1 PRISMA 2020 Checklist. Suppmentary Materials File S2 Systematic Review Protocol. Suppmentary Materials File S3 Systematic Review Search Strategy.
