# Supplementary material for: Current Evidence and Emerging Directions in Endometriosis Pharmacotherapy: A Systematic Review of Recent Advances
Source: J Clin Med. 2026 Sep 17;15(18):7238. doi: 10.3390/jcm15187238 (PMC13607422; doi:10.3390/jcm15187238)
Supplement: Supplementary file 1 [file jcm-15-07238-s001.zip › Suppmentary Materials File S2 Systematic Review Protocol.pdf]

Systematic Review Protocol

Title of the review	Current Evidence and Emerging Directions in Endometriosis Pharmacotherapy: A Systematic Review of Recent Advances
First reviewer	Miss Zuzanna Betiuk[1]
Team of reviewers	Martyna Bartoszczyk[1], Martyna Błażyca[1], Katarzyna Kutyła[1], Natalia Gierulska[1]
Supervisor/Project PI	prof. Rafał Tarkowski[2,] dr Krzysztof Kułak[2]
Clinical Portfolio Group	1. Students' Scientific Association at the 1st Chair and Department of Oncological Gynecology and Gynecology, Medical University of Lublin<br>2. 1st Chair and Department of Oncological Gynecology and Gynecology, Medical University of Lublin, Staszica 16 St., 20-081 Lublin, Poland
Protocol Version	1.2
Date of Finalization	29.09.2025
Date of Latest Revision	01.12.2025
PROSPERO Registration Status	Pending
Funding and Conflicts of Interest (CoI)	This systematic review is not supported by any external funding or pharmaceutical companies. The project is conducted within the framework of the Students' Scientific Association at the 1st Chair and Department of Oncological Gynecology and Gynecology at Medical University of Lublin.<br>The authors declare no conflicts of interest. None of the authors have financial or non-financial relationships with any pharmaceutical companies or commercial entities that could be perceived to influence the conduct or reporting of this review.

1. Background to review

Endometriosis is a chronic inflammatory condition affecting almost 190 million women of reproductive age worldwide, associated with severe chronic pain and a significantly reduced quality of life. While surgical intervention is a common option, many patients seek to avoid invasive procedures, making the continuous development and optimization of pharmacological treatments a clinical priority. Currently, there is a need to evaluate the long-term safety of established gold standards, while simultaneously exploring emerging therapies. This systematic review aims to synthesize the latest evidence on new GnRH antagonists, immunotherapy, and antioxidant. By addressing both the optimization of current protocols and the potential of non-hormonal directions, this study seeks to provide a comprehensive update that supports personalized, non-invasive management strategies for endometriosis.	
Comprehensive Review Question	What is the current state of knowledge in endometriosis pharmacotherapy, regarding the clinical efficacy and safety of progestins, GnRH modulators (antagonists and agonists), and drug combinations (including add-back therapy), and what are the emerging perspectives on immunotherapy for endometriosis treatment?
SR overview<br><br>A systematic review focusing on current pharmacological strategies and emerging therapeutic directions in endometriosis management.	
Research Gap	Although there are various pharmacological options for treating endometriosis, there is a lack of an up-to-date, systematic synthesis that would include information on the long-term results of using progestogens or GnRH modulators and the latest clinical discoveries on non-hormonal methods, such as immunotherapy.

2. Specific objectives
1. To evaluate the clinical efficacy, safety, and impact on fertility of progestins, with a specific focus on long-term dienogest therapy.<br>2. To summarize recent clinical findings on GnRH antagonists including their therapeutic roles and side-effect profiles.<br>3. To assess the integration of drug combinations and hormone add-back therapies in current endometriosis management protocols.<br>4. To identify emerging therapeutic directions, including antioxidants and immunotherapy agents and their potential in future endometriosis treatment.

3. a) Criteria for including studies in the review

I. Population, or participants and conditions of interest	Premenopausal women aged 15–50 years with diagnonsis of endometriosis.<br>Supplementary populations will also be included for the purpose of preliminary assessment and exploratory synthesis:<br>1. Healthy human volunteers (for pharmacological intervention)<br>2. Animal models (for early-stage experimental studies related to the mechanism of action or efficacy).<br>These supplementary populations will be used for a qualitative, exploratory synthesis of the drug's mechanism or future research directions and will be excluded from the main synthesis of clinical efficacy and patient-reported outcomes.
II. Interventions or exposures	The review will include studies assessing the efficacy and safety of the following pharmacological interventions used in the treatment of endometriosis, categorized by the review's thematic structure: Progestins (e.g., Dienogest), GnRH Antagonists (e.g., Relugolix, Elagolix), GnRH Agonists (e.g., leuprolide), drug combinations (including hormone add-back therapies) and immunotherapy agents currently being explored for endometriosis treatment. Studies on immunotherapy will be included for the purpose of a qualitative, exploratory synthesis on new research perspectives, but will be excluded from the main synthesis of clinical efficacy and patient-reported outcomes for approved standard-of-care agents.
III. Comparisons or control groups	The review will include studies utilizing both non-active and active comparator arms:<br>• Non-Active Control Groups (Placebo): This includes comparisons against Placebo in Randomized Controlled Trials (RCTs) or No Treatment control groups.<br>• Active Control Groups: This includes comparisons against any current standard pharmacological treatments for endometriosis (e.g., Dienogest, other Progestins, hormonal contraceptives) which will be grouped under the Usual Care tag.<br>• Non-Comparative Studies: Single-arm, non-comparative studies (e.g., Open-Label Extension

	studies, cohort studies) will be included. These are crucial for collecting long-term safety and adverse event data (e.g., VTE, BMD), or for providing exploratory data.
IV. Outcomes of interest	Clinically Meaningful Pain Response: The proportion of patients achieving a clinically meaningful treatment response for endometriosis-associated pain (dysmenorrhea, NMPP, dyspareunia). The response will be defined hierarchically based on the data reported in the primary studies (Priority 1: ≥50% reduction in VAS/NRS; Priority 2: ≥2 points on NRS, or other established clinically relevant improvement). Data will be measured at 3, 6, and 12 months from baseline, and at the End of Study (EOS).<br>Pain Interference: The level of interference with daily activity and sleep, measured by validated scales (e.g., Brief Pain Inventory (BPI)). Data will be measured at 3, 6, and 12 months from baseline, and at the EOS.<br>Health-Related Quality of Life (QoL): Change from baseline in QoL scores, measured by validated instruments (e.g., EHP-30 questionnaire). Data will be measured at 6 and 12 months from baseline, and at the EOS.<br>Endometrioma Size: Change from baseline in endometriosis lesion volume (or diameter), measured by ultrasound. Data will be measured at 6 and 12 months from baseline.<br>Comprehensive Safety Profile: Incidence of all Treatment-Emergent Adverse Events (TEAEs), Serious Adverse Events (SAEs), and rates of discontinuation due to AEs. This includes specific safety concerns: Venous Thromboembolism (VTE), changes in Bone Mineral Density (BMD) at the lumbar spine and femoral neck (measured at 3, 6, and 12 months and at the EOS), changes in vital signs, weight, ECG, and clinical laboratory tests. Data will be measured at all reported time points.<br>Long-term Safety: Incidence of endometrial cancer and tubo-ovarian cancer. Data will be measured at the EOS.<br>Exploratory Markers: Changes in inflammatory biomarkers, angiogenic pathway parameters. Data will be measured at all reported time points.

V. Setting	Studies will be included only if participants were recruited in a formal clinical setting (e.g., gynecology clinics or hospitals). Although the administration of the intervention and patient reporting of outcomes (e.g., NMPP or dysmenorrhea score) may have occurred outside the clinic (e.g., in the patient's home), the study must have been conducted under professional clinical supervision and monitoring to ensure data integrity and patient safety. We will not apply geographical restrictions.
VI. Study designs	We will include prospective and retrospective cohort studies and RCT, but we will also include data on long-term safety and efficacy from the corresponding Long-term Open-Label Extension (OLE) studies that follow the included RCTs.<br>Randomized pilot studies, feasibility studies, case report, animal studies and in vitro studies will also be included, but their data will only be used for an exploratory narrative synthesis (chapter on new perspectives and hopes) and will be excluded from any quantitative analysis due to insufficient statistical power.
3. b) Criteria for excluding studies not covered in inclusion criteria	
We will exclude studies involving male participants, postmenopausal women, participants under 15 years of age, pregnant and breastfeeding women, participants with active malignancy (particularly hormone-dependent), current or history of thromboembolic disorders, uncontrolled or severe cardiovascular conditions (including severe hypertension and cardiac disease), active liver failure or other significant liver diseases that may compromise drug metabolism.<br>We will exclude studies where the intervention is non-pharmacological, including surgical treatments (laparoscopy, hysterectomy, etc.), urogynecological physical therapy and other forms of physical or manual therapy, psychotherapy and sexological therapy, Complementary and Alternative Medicine (CAM), including herbal remedies, dietary supplements, essential oils, acupuncture, or homeopathy.<br>We will exclude studies where the comparator group involves the following:<br>comparisons against non-pharmacological interventions such as surgical procedures, physical therapy, or alternative medicine;<br>comparisons against unapproved or experimental agents that are not currently standard of care and whose efficacy is highly speculative;<br>We will exclude meta-analyses, systematic reviews, narrative reviews, scoping reviews, literature reviews, studies not published in English, conference abstracts, expert opinions.	

4. Search methods	
Electronic databases	MEDLINE, PubMed from 2019/12/01 to 2025/12/01<br>In addition to the primary databases (MEDLINE) searching will be performed in Google Scholar to identify grey literature and broaden the scope of the search, particularly for non-indexed exploratory studies. We will also search trial registers (e.g., ClinicalTrials.gov).
Other methods used for identifying relevant research	Reference checking (e.g., citation chasing) will be performed on all studies included at the full-text stage. We will check the reference lists of included studies to identify additional relevant reports. No researchers or experts will be contacted.
Journals hand searched	No manual journal hand-searching will be conducted.
Full Search Strategy	(("Endometriosis"[MeSH]) OR (endometriosis[tiab] OR endometrioma*[tiab] OR endometriotic*[tiab] OR "deep infiltrating endometriosis"[tiab])) AND (("Drug Therapy"[MeSH] OR Immunotherapy [MeSH] OR Progestins [MeSH] OR "Hormone Antagonists"[MeSH] OR "Aromatase Inhibitors"[MeSH]) OR (Pharmacotherapy[tiab] OR drug[tiab] OR medicat*[tiab] OR antibod*[tiab] OR interlekin*[tiab] OR astaxanthin[tiab] OR anakinra[tiab] OR bevacizumab*[tiab] OR cabergoline[tiab] OR "dopamine receptor D2 agonists"[tiab] OR progestagen*[tiab] OR progestin*[tiab] OR gestagen*[tiab] OR dienogest[tiab] OR drospirenone[tiab] OR estetrol[tiab] OR "GnRH antagonist*"[tiab] OR elagolix[tiab] OR relugolix[tiab] OR letrozole[tiab] OR anastrozole[tiab])) AND ((Pain[MeSH] OR "Adverse Drug Reaction"[MeSH] OR "Quality of Life"[MeSH] OR Neoplasms[MeSH] OR Fertility[MeSH] OR Inflammation[MeSH]) OR (pain[tiab] OR dysmenorrhea[tiab] OR dyspareunia[tiab] OR "analgesic/opioid use"[tiab] OR "non menstrual pelvic pain"[tiab] OR "endometriosis-associated pain"[tiab] OR "quality of life"[tiab] OR QoL[tiab] OR recurren*[tiab] OR relapse*[tiab] OR "side effect*"[tiab] OR tolerab*[tiab] OR safe*[tiab] OR oncol*[tiab] OR malignan*[tiab] OR cancer[tiab] OR tumor*[tiab] OR fertil*[tiab] OR infertil*[tiab] OR regress*[tiab] OR redu*[tiab] OR inflamm*[tiab] OR recurren*[tiab])) AND ((Randomized Controlled Trial[pt] OR Cohort Study[pt] OR Prospective Studies[pt] OR Retrospective Studies[pt] OR "Open-Label Extension"[tiab] OR Journal Article[pt])) NOT ((Systematic Review[pt] OR Meta-Analysis[pt] OR Review[pt] OR Letter[pt] OR

	Editorial[pt] OR Comment[pt] OR "expert opinion"[tiab] OR "conference abstract"[tiab]))

5. Methods of review	
Details of methods	Four reviewers will be involved in the screening and selection process. Titles and abstracts will be screened independently. The full text will then be retrieved and assessed for eligibility. Any disagreements between reviewers will be resolved through consensus or, if necessary, by a third senior reviewer from the team.
Quality assessment	The risk of bias for randomized controlled trials will be assessed using the Cochrane Risk of Bias tool (RoB 2). For observational cohort studies and other non-randomized studies of interventions, the ROBINS-I tool will be applied. This approach ensures a rigorous and consistent evaluation of potential biases across different study designs. Each study will be assessed independently by at least two reviewers.
Data extraction	Data will be extracted using a Excel.. The following information will be extracted: study characteristics (author, year, study design), participant demographics, intervention details (type of drug, dosage, duration of treatment), and outcomes (pain scores, safety data, impact on fertility).
Narrative synthesis	A narrative synthesis will be conducted to summarize findings. Evidence will be grouped by intervention type (e.g., progestogens, GnRH antagonists/agonists, immunotherapy). The synthesis will focus on clinical efficacy, safety profiles, and emerging therapeutic trends, supported by summary tables and descriptive text.
Meta-analysis	Due to anticipated clinical and methodological heterogeneity among the included studies, a meta-analysis is not primarily planned. It will only be considered if sufficient homogenous data are available.
Grading evidence	GRADE

6. Presentation of results	
Additional material	PRISMA Flow Diagram; Table of Included Study Characteristics; Contextual Summary Table: Pharmacological Dosing of the described drugs, Comparative Tables of Study Results and Methodology; Contextual Summary Figure: Pharmacological Management Recommendations for Endometriosis

Outputs from review	Scientific manuscript submitted for publication in a peer-reviewed medical journal focused on gynecology or pharmacology.

7. Timeline for review – when do you aim to complete each stage of the review	
Protocol	1 month
Literature searching	2-3 weeks
Quality appraisal	2-3 weeks
Data extraction	3-4 weeks
Synthesis	1 months
Writing up	6-8 weeks
