# Supplementary material for: Current Evidence and Emerging Directions in Endometriosis Pharmacotherapy: A Systematic Review of Recent Advances
Source: J Clin Med. 2026 Sep 17;15(18):7238. doi: 10.3390/jcm15187238 (PMC13607422; doi:10.3390/jcm15187238)
Supplement: Supplementary file 1 [file jcm-15-07238-s001.zip › Suppmentary Materials File S3 Systematic Review Search Strategy.pdf]

Systematic Review Search Strategy

Review title: New Perspectives and Directions in Endometriosis Pharmacotherapy: A Systematic Review of the Latest Literature

Medline (PubMed)	
1.	Endometriosis [MeSH]
2.	Endometriosis[tiab] OR endometrioma*[tiab] OR endometriotic*[tiab] OR “deep infiltrating endometriosis”[tiab]
3.	#1 OR #2
4.	“Drug therapy” [MeSH] OR Immunotherapy [MeSH] OR Progestins [MeSH] or OR “Hormone Antagonists”[MeSH] OR “Aromatase Inhibitors”[MeSH]
5.	Pharmacotherapy[tiab] OR drug[tiab] OR medicat*[tiab] OR antibod*[tiab] OR interlekin*[tiab] OR astaxanthin[tiab] OR anakinra[tiab] OR bevacizumab*[tiab] OR cabergoline[tiab] OR “dopamine receptor D2 agonists” OR progestagen*[tiab] OR progestin*[tiab] OR gestagen*[tiab] OR dienogest[tiab] OR drospirenone[tiab] OR estetrol[tiab] OR "GnRH antagonist* OR elagolix[tiab] OR relugolix[tiab] OR letrozole[tiab] or anastrozole[tiab]
6.	#4 OR # 5
7.	Pain[MeSH] OR "Adverse Drug Reaction" OR "Quality of Life"[MeSH] OR Neoplasms[MeSH] OR Fertility[MeSH] OR Inflammation[MeSH]
8.	pain[tiab] OR dysmenorrhea[tiab] OR dyspareunia[tiab] OR “analgesic/opioid use”[tiab] OR “non menstrual pelvic pain”[tiab] OR “endometriosis-associated pain”[tiab] OR "quality of life"[tiab] OR QoL[tiab] OR recurren[tiab] OR relapse[tiab] OR "side effect*"[tiab] OR tolerab*[tiab] OR safe*[tiab] R oncol*[tiab] OR malignan*[tiab] OR cancer[tiab] OR tumor*[tiab] OR fertil*[tiab] OR infertil*[tiab] OR regress*[tiab] OR redu*[tiab] OR inflamm*[tiab] OR recurren*[tiab]
9.	#7 OR #8
10.	Randomized Controlled Trial[pt] OR Cohort Study[pt] OR Prospective Studies[pt] OR Retrospective Studies[pt] OR "Open-Label Extension"[tiab] OR Journal Article[pt]
11.	Systematic Review[pt] OR Meta-Analysis[pt] OR Review[pt] OR Letter[pt] OR Editorial[pt] OR Comment[pt] OR "expert opinion"[tiab] OR "conference abstract"[tiab]
12.	#3 AND #6 AND #9 AND #10 NOT #11

(("Endometriosis"[MeSH]) OR (endometriosis[tiab] OR endometrioma*[tiab] OR endometriotic*[tiab] OR "deep infiltrating endometriosis"[tiab])) AND (("Drug Therapy"[MeSH] OR Immunotherapy [MeSH] OR Progestins [MeSH] OR "Hormone Antagonists"[MeSH] OR "Aromatase Inhibitors"[MeSH]) OR (Pharmacotherapy[tiab] OR drug[tiab] OR medicat*[tiab] OR antibod*[tiab] OR interlekin*[tiab] OR astaxanthin[tiab] OR anakinra[tiab] OR bevacizumab*[tiab] OR cabergoline[tiab] OR "dopamine receptor D2 agonists"[tiab] OR progestagen*[tiab] OR progestin*[tiab] OR gestagen*[tiab] OR dienogest[tiab] OR drospirenone[tiab] OR estetrol[tiab] OR "GnRH antagonist*"[tiab] OR elagolix[tiab] OR relugolix[tiab] OR letrozole[tiab] OR anastrozole[tiab])) AND ((Pain[MeSH] OR "Adverse Drug Reaction"[MeSH] OR "Quality of Life"[MeSH] OR

Neoplasms[MeSH] OR Fertility[MeSH] OR Inflammation[MeSH]) OR (pain[tiab] OR dysmenorrhea[tiab] OR dyspareunia[tiab] OR "analgesic/opioid use"[tiab] OR "non menstrual pelvic pain"[tiab] OR "endometriosis-associated pain"[tiab] OR "quality of life"[tiab] OR QoL[tiab] OR recurren*[tiab] OR relapse*[tiab] OR "side effect*"[tiab] OR tolerab*[tiab] OR safe*[tiab] OR oncol*[tiab] OR malignan*[tiab] OR cancer[tiab] OR tumor*[tiab] OR fertil*[tiab] OR infertil*[tiab] OR regress*[tiab] OR redu*[tiab] OR inflamm*[tiab] OR recurren*[tiab])) AND ((Randomized Controlled Trial[pt] OR Cohort Study[pt] OR Prospective Studies[pt] OR Retrospective Studies[pt] OR "Open-Label Extension"[tiab] OR Journal Article[pt])) NOT ((Systematic Review[pt] OR Meta-Analysis[pt] OR Review[pt] OR Letter[pt] OR Editorial[pt] OR Comment[pt] OR "expert opinion"[tiab] OR "conference abstract"[tiab]))
