# Supplementary material for: Current Evidence and Emerging Directions in Endometriosis Pharmacotherapy: A Systematic Review of Recent Advances
Source: J Clin Med. 2026 Sep 17;15(18):7238. doi: 10.3390/jcm15187238 (PMC13607422; doi:10.3390/jcm15187238)
Supplement: Supplementary file 1 [file jcm-15-07238-s001.zip › Table S1 Characteristics of the included studies.pdf]

Table S1 Characteristics of the included studies

References	*Study type*	*Population*	*Dates*	*Intervention*	*Controls*	*Funding*	*RoB score*	*Primary outcome*	*Secondary outcome*	*Safety outcome*	*GRADE Certainty*	*GRADE Explanation*
Niakan et al. (2021) (9)	Double-blind RCT	90 women aged 18-45 years with endometriosis confirmed by laparoscopic surgery	03.2018-03.2020	Daily DNG (2 mg tablet, *Visanne*) or OCPs for 12 weeks	Placebo (N=36)	Iran University of Medical Sciences	Some concerns	**Pain** (6 months): Significant reduction in pelvic pain and dyspareunia in both DNG and COCP groups compared to placebo ($p < 0.05$).	**QoL** (6 months): Significant improvement in DNG group (Mean Score Increase: +22.00) vs. placebo (+6.45) and COCP (+23.45) group vs. placebo (+6.45) Significant difference vs. placebo was confirmed for both DNG ($p < 0.001$) and COCPs ($p = 0.004$). Positive effect observed for dysuria and dyschezia.	Safety: Well tolerated; no significant difference in AE rates or discontinuation vs. placebo/COCPs. Specific side effects: No significant difference between DNG and COCPs (e.g., vaginal bleeding: OR 0.88; 95% CI 0.39–1.96; p=0.75). Other AEs: p>0.05.	For pelvic pain, dyspareunia and QoL: moderate ⊕⊕⊕◯	While the study was well-designed and blinded, the results are limited by a small sample size.
Yurtkal et al. (2024) (10)	Prospective cohort study	60 women aged 18-45 years diagnosed with endometriosis	NR	Cyclic DNG (*Visanne*)(N=20); 0.03 mg ethinylestradiol combined with 2 mg DNG (*Dienille*)(N=20); estradiol valerate combined with 2 mg DNG	No control group	No financial support	Moderate	**Pelvic Pain** (VAS): All three treatment groups showed a statistically significant reduction in VAS scores compared to baseline. No statistically significant difference in efficacy between	**AMH levels**: there was no significant discrepancy between the drug factions in terms of AMH at the initial examination and AMH levels in the 6th month after drug utilization (P >0.05). **CA-125 levels**: there was no significant discrepancy between the drug factions in the	NR	For endometriosis pain: very low ⊕◯◯◯	As an observational cohort study, the findings are susceptible to selection bias and confounding factors.

				(Qlarista)(N =20)				groups; pain reduction was consistent and similar across all arms. Specific mean VAS scores and percentage reductions were not reported.	initial Ca-125 and post drug CA-125 levels (P>0.05).			
Caruso s et al. (2022) (11)	Randomized, comparative study	197 women aged 18-40 years with endometriosis	NR	COC containing 1.5 mg 17β-estradiol and 2.5 mg E2/NOMAC (N=99) or 2 mg daily DNG oral progestin (N=98)	No control group	No financial support	Moderate	**Chronic Pelvic Pain** (VAS): Significant reduction in both groups at 6 months (NOMAC/E2: –5.2 ± 2.1; DNG: –5.4 ± 2.3). No statistically significant difference between groups (p > 0.05). Clinically meaningful pain reduction (≥30% VAS decrease) achieved by 78% (NOMAC/E2) vs. 81% (DNG) of patients.	QoL (SF-36, 6–12m): Significant improvement in both (NOMAC/E2: +12.5±4.3; DNG: +13.1±4.6). Sexual Function: Greater improvement in DNG. Dysmenorrhea: Comparable (NOMAC/E2: –4.8±2.0; DNG: –4.9±2.2). Dyspareunia: Comparable (NOMAC/E2: –3.7±1.8; DNG: –3.8±1.9).	AEs are reported in 13.3% of women in the NOMAC/E2 group and 16.7% in the DNG group (p = 0.65). The most common AEs are breakthrough bleeding (NOMAC/E2: 6.7%; DNG: 10%) and headache (NOMAC/E2: 3.3%, DNG: 6.7%). No SAEs or treatment discontinuations due to AEs were reported in either group.	For chronic pelvic pain, QoL and sexual function: Low ⊕⊕◯◯	The final rating is low due to downgraded indirectness and imprecision - the study cohort was relatively small, what lead to wider confidence intervals.

Alithan et al. (2025) (13)	Retrospective cohort study	180 women aged 29-40 years with EAP	03.2018-03.2023	Oral DNG 2 mg once daily (N=180)	No control group	No financial support	Moderate	Follow-up: N=180 (T0/T1); 12m: 104 (57.8%); 18m: 65 (36.1%); 24m: 17 (9.4%); 30m: 4 (2.2%). Endometrioma Loc.: Diff. between groups (p<0.001); G1 higher right-side (52.6% vs 29.8%; p=0.002), G2 higher bilateral (39.8% vs 13.2%; p<0.001). Dysmenorrhea (VAS): Higher in G2 at T0/T1 (p≤0.02). Decrease in both at T1 (p<0.001); intergroup difference in reduction (p=0.003).	AEs: Libido lower in G2 (10.5% vs 45.2%; p<0.001); weight gain higher in G1 (88.2% vs 76%; p=0.02). Spotting/headache: NS (p=0.3/0.07). BMI: G1 < G2 (22 vs 23; p<0.001). Dyspareunia (VAS): Higher in G2 at T0/T1 (p≤0.007). Reduction in both at T1 (p<0.001); G1 reduction greater (p=0.001). NMPP (VAS): Higher in G2 at T0/T1 (p<0.001). Both improved at T1 (p<0.001); no inter-group difference in variation (p=0.5).	No SEAs were reported, and the overall tolerability is acceptable. The study do not report specific rates for other AEs.	For reduction in endometrioma size and pain: very low ⊕◯◯◯	Downgraded for RoB and Imprecision. Patients were not randomized.
La Torre et al. (2024) (14)	Cohort study	114 women aged 18-45 years with endometriosis undergoing	NR	Oral DNG for long-term management (N=114)	No control group	No financial support	Low	**Long-term Efficacy (36 months)**: Significant VAS reduction across all	At 12 months, 81% of patients were continuing the DNG treatment, with a significant reduction of dysmenorrhea,	AEs: Spotting, low libido, vaginal dryness, and mood disorders common in 1st year. Generally	For long-term pain relief, safety and tolerability and treatment	As a retrospective observational study without a control

		long-term treatment						phenotypes, most pronounced in deep infiltrating endometriosis. 73% of patients adhere to the treatment. Amenorrhea rate increased from 77% (12 months) to 93% (36 months)	dyspareunia, dyschezia, dysuria and chronic pelvic pain. Of the 19% that discontinued the treatment: 62% was due to spotting, reduced sexual drive, vaginal dryness, and mood disorders.	mild; no SAEs. Long-term tolerability: Favorable; most 1-year completers remained on therapy at 36m.	persistence: Very low ⊕◯◯◯	group, it cannot definitively prove causality.
Park Y et al. (2025) (15)	Prospective study	101 women diagnosed with endometriosis by using imaging techniques or postoperative pathological tests	04.2019-04.2021	Oral DNG 2 mg daily for 6 months (N=97→30)	NETA (5 mg/day) + placebo (twice weekly) for 6 months (N =4→3)	No financial support	Serious	**Reduction of menstrual pain** (VAS): decrease from 5.75 to 1.40; **changes in psychiatric variables** (CES-D: increase from 9.1 to 13.2 and STAI-S: increase from 35.6 to 36.2, STAI-T: increase from 34.5 to 37.1), no statistical significance in risk factors associated with depressive symptoms	Reduction of the size of ovarian endometrioma: decreased by −24.6 ± 15.7 % after DNG treatment, decreased by −98.0 % ± 5.6 % when treated with DNG after surgery	Clinically significant depressive symptoms	For reduction of menstrual pain, of the size of ovarian endometrioma, changes in psychiatric variables and risk factors associated with depressive symptoms: Very Low ⊕◯◯◯	The evidence was downgraded due to serious attrition bias (only 30 out of 97 patients completed the follow-up), Rob (small control group and blinding), and imprecision from the small final cohort.

								among DNG users				
Kim et al. (2021) (17)	Retrospective cohort study	44 women aged 18-45 years who underwent laparoscopic surgery for endometrioma and subsequently received DNG	07.2013-12.2018	DNG 2 mg daily (N=44)	No control group	No financial support	Moderate	**BMD (3 years)**: Lumbar spine: –4.4%; Femoral neck: –3.6% (n=44). Most loss occurred in Year 1 (–2.4% at lumbar spine), with the rate of reduction slowing in subsequent years.	No predictive factors for BMD reduction during the first year of treatment with DNG were found	NR	For BMD and recurrence rate: Very Low ⊕◯◯◯	While the study utilizes objective DXA measurements, its retrospective design means it cannot fully account for all variables influencing bone health.
Jhang H et al. (2025) (18)	Retrospective cohort study	4,647 women aged 20–49 years with endometriosis	01.2021-12.2023	Exposure to DNG for at least 6 months (N=4647)	GnRH agonist users (N=181587)	No financial support	Moderate	Breast Cancer Risk (DNG): Reduced risk with 0.5–1.5 years of use (aHR 0.72; 95% CI 0.53–0.99). Results for durations >1.5 years were inconsistent.	Cancer Risk (DNG vs. GnRH agonists): No increased risk observed for: Breast cancer: aHR 1.01 (95% CI 0.53–1.37); Endometrial cancer: aHR 0.84 (95% CI 0.40–1.77); Tubo-ovarian cancer: aHR 0.92 (95% CI 0.30–2.80).	NR	For risk of breast cancer, endometrial cancer, tubo-ovarian cancer: Very Low ⊕◯◯◯	Non-randomized design; limited by residual confounding. Despite large nationwide sample and robust weighting, inherent observational nature and risk of unmeasured bias preclude higher

												certainty ratings per GRADE criteria.
Barra et al. (2020) (20)	Retrospective cohort study	151 women (18–45 yrs) with imaging-confirmed endometriosis and 1 failed IVF cycle/embryo transfer history	NR	DNG 2 mg daily for 3 months prior to IVF	Control group consisted of 88 women who underwent IVF without prior hormonal treatment	No financial support	Moderate	Cumulative IVF Success (DNG vs. Control): Implantation rate: 39.7% vs. 23.9% (p=0.049). Clinical pregnancy: 33.3% vs. 18.2% (p=0.037). Live birth rate: 28.6% vs. 14.8% (p=0.043).	Endometriomas ≥4 cm (DNG vs. Control): Significant reduction in endometrioma diameter (p < 0.001). Significantly higher number of retrieved oocytes (p = 0.031), 2PN embryos (p = 0.039), and blastocysts (p = 0.005) in the DNG group.	NR	For clinical pregnancy rate, number of oocytes and implantation rate: Very Low ⊕◯◯◯	Downgraded for imprecision: The sample size (n=102) is below the Optimal Information Size (OIS). Low event rates in fertility outcomes result in wide 95% CIs and potential instability of effect estimates.
Tamura et al. (2019) (21)	Prospective cohort study	68 infertile women aged 18-45 years with Stage III or IV endometriosis and ovarian endometrial cysts less than 4 cm	NR	DNG 2 mg orally daily for 12 weeks prior to IVF (N=33)	Standard controlled ovarian hyperstimulation with the GnRH agonist (N=35)	Mochida Pharmaceutical Co. Ltd., Tokyo, Japan	Moderate	IVF Outcomes (DNG vs. Control): Implantation rate: 18.2% vs. 22.2% (p=NS). Growing follicles: 6.2 vs. 8.7 (p<0.05). Retrieved oocytes: 5.7 vs. 8.1 (p<0.05).	Abortion rate: 33.3% (DNG) vs. 18.2% (Control), p=NS. No significant reduction in inflammatory cytokines or oxidative stress markers in follicular fluid.	SAEs=0 study do not provide specific rates of AEs or discontinuation.	For pregnancy rate for transfer, numbers of oocyted recieved and cumulative pregnancy rate: Very Low ⊕◯◯◯	Limited by non-randomized, retrospective design and susceptibility to unmeasured confounding.

Mehdizadeh K et al. (2022) (60)	Double-blind, pilot RCT	108 women aged 18–45 years with laparoscopically confirmed stage IV endometriosis	03.2018 - 03.2020	DNG 2 mg orally daily for 6 months (N= 36→30)	1. Active Control: COCP containing 30 µg EE+ 0.3 mg levonorgestrel daily (N=36→30) 2.Placebo Control: placebo daily (N=36→29)	Iran University of Medical Sciences	Some concerns	**Change in pain scores** (VAS) from baseline to 6 months. **Pelvic Pain:** DNG -5.39; COCP -5.79; Placebo -3.14 (p=0.026) **Dyspareunia**: DNG -2.14; COCP -2.86; Placebo -0.48 (p=0.040) (Active treatments superior to placebo, p<0.05; no significant difference between DNG and COCP)	**QOL** (WHOQOL-BREF): Significant mean score increase for DNG (+22.00) and COCP (+23.45) vs placebo (+6.45; p=0.001). **Pain Recurrence** (VAS > 5): Reported in 6.6% of patients in both active groups vs 20.7% in placebo.	Discontinuation rate: 2.8% (due to AEs). Common AEs: Spotting (20% DNG vs 13.3% COCP), Headache/Nausea /Hair loss (13.3% DNG vs 20% COCP). No significant differences between groups.	For change in pain scores, QoL: Moderate ⊕⊕⊕◯ For Pain Recurrence: Low ⊕⊕◯◯	Moderate (Pain, QoL): Downgraded for serious imprecision (N=89 analyzed, below optimal information size). Low (Pain recurrence): Downgraded for very serious imprecision; extremely low event rate (N=2/arm) and wide CIs prevent precise estimation.
Ma Y et al. (2024) (12)	Retrospective cohort study	154 patients who underwent laparoscopic cystectomy for ovarian endometriosis	01.2019 – 12.2022	3 to 4 injections of GnRH-a (3,75 mg Leuprorelin acetate) post-surgery, followed by DNG	DNG 2 mg orally daily (N=70→70)	No financial support	Serious	Irregular bleeding: 14.3% vs. 42.9% (p<0.05). Pelvic pain (VAS, 3m): 1.35±0.50 vs. 1.79±0.61. Serum CA125 (3m):	The recurrence rate - lower in the intervention group (2.38%) compared to the control group (7.14%) - the difference was not statistically significant (p>0.05)	Perimenopausal symptoms (e.g., hot flashes): 16 cases (19.05%) in intervention vs. 2 cases (2.86%) in control (p<0.05)	For incidence of irregular vaginal bleeding, pelvic pain, CA125 levels, recurrence rate: Very	Downgraded for indirectness (short follow-up) and imprecision (rare events). No upgrade

				therapy (N=84→84)				19.86±5.34 vs. 26.43±8.12 U/mL.			low ⊕◯◯◯	criteria met.
Giudice LC et al. (2022) SPIRIT 1 (24)	Phase 3, double-blind RCT	638 premenopausal women aged 18–50 years with endometriosis or with moderate to severe dysmenorrhea and NMPP	12.2017-12.2019	Once daily oral relugolix CT for 24 weeks (N=212→181) or delayed relugolix CT (N=213→182)	Placebo (N=213→174)	Myovant Sciences, Bayer, AbbVie, Roche, and others	Low	Clinically meaningful pain reduction (NRS reduction ≥2.8 for dysmenorrhea and ≥2.1 for NMPP) with no increase in analgesic use at Week 24 Dysmenorrhea: Relugolix CT 75% (158 of 212) vs Placebo 27% (57 of 212) (p<0.0001) NMPP: Relugolix CT 58% (124 of 212) vs Placebo 40% (84 of 212) (p<0.0001)	Week 24: EHP-30 (pain): Relugolix: –33.8 vs placebo: –18.7 (p<0.0001) Pelvic pain (NRS): –3.1 vs –1.9 (p<0.0001) Dyspareunia (NRS): –2.4 vs –1.7 (p=0.0149) Analgesic-free: 56% vs 31% (p<0.0001) Opioid-free: 86% vs 76% (p=0.0005)	TEAEs: 71% vs 66% (Common: headache, nasopharyngitis, hot flushes). SAEs: 3 vs 5 (no deaths). BMD (Δ): –0.70% vs +0.21%. Endometrial biopsy: No hyperplasia/malignancy.	For dysmenorrhea, NMPP, EHP-30 pain domain, overall pelvic pain, dyspareunia, analgesic-free and opioid-free status, TEAEs, SAEs and BMD loss: High ⊕⊕⊕⊕	High certainty from a large RCT. No concerns in bias, consistency, or precision; results are robust with narrow CIs and high power.
Giudice LC et al. (2022) SPIRIT 2 (24)	Phase 3, double-blind RCT	623 premenopausal women aged 18–50 with endometriosis or with moderate to severe dysmenorr	10.2017-11.2019	Once daily oral relugolix CT (N=208→174) or delayed relugolix CT for 24 weeks	Placebo (N=208→169)	Myovant Sciences, Bayer, AbbVie, Roche, and others	Low	Clinically meaningful pain reduction (NRS reduction ≥2.8 for dysmenorrhea and ≥2.1 for NMPP) with no increase in	Week 24: EHP-30: –32.2 vs –19.9 (p<0.0001) Pelvic pain (NRS): –2.9 vs –2.0 (p<0.0001) Dyspareunia (NRS): –2.4 vs –1.9 (p=0.0371)	TEAEs: 81% vs 75% (Common: headache, nasopharyngitis, hot flushes). SAEs: 9 vs 4 (no deaths). BMD loss (Δ): –0.78% vs +0.02%. Endometrial biopsy: No	For dysmenorrhea, NMPP, overall pelvic pain. dyspareunia reduction, EHP-30	High certainty from a large RCT. No concerns in bias, consistency, or precision; results are

		hea and NMPP		(N=207→165)				analgesic use at Week 24: Dysmenorrhea: Relugolix CT 75% (155 of 206) vs Placebo 30% (62 of 204) (p<0.0001) NMPP: Relugolix CT 66% (136 of 206) vs Placebo 43% (87 of 204) (p<0.0001)	Analgesic-free: 54% vs 32% (p<0.0001) Opioid-free: 82% vs 66% (p=0.0005)	hyperplasia/malignancy.	pain domain, analgesic-free and opioid-free status, TEAEs, SAEs and BMD loss: High ⨁⨁⨁⨁	robust with narrow CIs and high power.
Becker CM et al. (2024) (25)	Long-term open-label extension study	802 premenopausal women aged 18–50 with confirmed endometriosis and moderate to severe dysmenorrhea and NMPP who completed the 24-week pivotal studies (SPIRIT 1 and 2)	05.2018-01.2023	Once-daily oral relugolix CT for additional 80weeks (N=802→501)	No control group	Myovant Sciences GmbH	Moderate	Meaningful pain reduction (≥2.8 dysmenorrhea/≥2.1 NMPP, no increased analgesics): Week 52: Dysmenorrhea: 84.8% (CT) vs 82.2% (Delayed) vs 75.6% (placebo). NMPP: 73.6% vs 70.4% vs 68.0%. Week 104: Dysmenorrhea: 84.8% vs 83.0% vs 80.4%. NMPP: 75.8% vs	Week 104: EHP-30 pain domain score: 88.6% responders (defined as improvement of ≥20 points from baseline) Overall pelvic pain: 69.4% reduction from baseline Dyspareunia: 58.6% reduction from baseline Analgesic-free status: 75.1% Opioid-free status: 91%	TEAEs: 90.5% (726/802) Most common: headache, nasopharyngitis and hot flushes. SAEs: 41 BMD at Week 104: Mean change from baseline (Lumbar spine): –1.07% (95% CI: –1.33, –0.82) Clinically significant loss (>3%): 19.8% of patients. BMD remained stable from Week 24 to Week 104.	For dysmenorrhea, NMPP, overall pelvic pain and dyspareunia reduction, EHP-30 pain domain, analgesic-free and opioid-free status, TEAEs, SAEs and BMD: Low ⨁⨁◯◯	Downgraded due to open-label, single-arm design.

								71.7% vs 73.1%.				
Osuga Y et al. (2021)a (26)	Phase 2, double-blind RCT	487 Japanese premenopausal women aged 20-50 with confirmed endometriosis	12.2011 - 09.2013	Once daily oral relugolix 10 mg (N=103→101), relugolix 20 mg (N=100→92), relugolix 40 mg (N=103→101) or once monthly subcutaneous injection leuprorelin 3.75 mg (N=82→77) for 12 weeks	Placebo (N=99→97)	Takeda Pharmaceutical Company Limited	Low	Mean change in VAS (pelvic pain) 28 days before EOT:<br>Placebo: −3.8 mm<br>Relugolix 10 mg: −6.2 mm (p<0.05)<br>Relugolix 20 mg: −8.1 mm (p<0.05)<br>Relugolix 40 mg: −10.4 mm (p<0.0001)<br>Leuprorelin: −10.6 mm	Pelvic pain & dysmenorrhea decreased in a dose-response manner (from Month 1); Relugolix 40 mg comparable to leuprorelin. No clear trend for dyspareunia	TEAEs: placebo (71.1%) vs Relugolix 10/20/40mg (79.6%/89.0%/94.2%) vs Leuprorelin (91.3%). Common (>10%): Metrorrhagia, irregular menses, hot flush, nasopharyngitis, headache. SAEs: 1 (placebo); no deaths. BMD (Δ 12w): placebo: –0.1(1.7); Relugolix: –1.0(1.9)/–1.3(2.1)/–2.1(2.2); Leuprorelin: –2.2(1.7). Other: No clinically significant changes (vitals, weight, ECG, labs).	For mean change in VAS (pelvic pain) and dysmenorrhea: High ⊕⊕⊕⊕<br>For dyspareunia and SAEs BMD (mean % change from baseline), AEs: Moderate ⊕⊕⊕◯	High: Multicenter RCT; no RoB, inconsistency, or indirectness. Results strengthened by dose-response gradient<br>Moderate: Downgraded for imprecision: phase 2 sample size (n≈100/arm)
Osuga Y et al. (2021)b (27)	Phase 2, open-label, parallel-group extension RCT	397 Japanese premenopausal women aged 20-50 with endometriosis, who completed the preceding	03.2012-02.2014	Once daily oral relugolix 10 mg (N=84→79), relugolix 20 mg (N=78→76), relugolix 40 mg (N=89→88) or once	Placebo (N=77→68)	Takeda Pharmaceutical Company Limited	Some concerns	TEAEs:<br>Placebo: 81.4%;<br>Relugolix (10/20/40 mg): 86.4%/96.0%/95.1%;<br>Leuprorelin: 97.5%<br>SAEs: 7; no deaths reported	Mean Δ VAS [mm] (SD) at EOT:<br>Pelvic pain: placebo: –3.2(12.2); Relugolix 10/20/40mg: –6.8(10.6)/–9.0(11.8)/–11.9(11.3); Leuprorelin: –12.7(12.6).	Common TEAEs (≥10%): nasopharyngitis, headache, metrorrhagia, irregular menstruation, menorrhagia, oligomenorrhea, hyperhidrosis, and hot flush. Incidence: higher	For mean change in VAS (pelvic pain, dysmenorrhea) (24 weeks): High ⊕⊕⊕⊕<br>For dyspareuni	Moderate: downgraded for open-label design and imprecision; small extension sample (n≈90/arm)<br>High: supported

		12-week relugolix phase 2 study		monthly subcutaneous injection leuprorelin 3.75 mg (N=69→62) for additional 12 weeks				BMD mean % change from baseline (SD) at Week 24: Placebo: –0.2 (1.99); Relugolix (10/20/40 mg): –1.6 (2.34) / –2.6 (2.94) / –4.9 (2.91); Leuprorelin: –4.4 (2.16)<br>No clinically significant changes in vital signs, ECG, or lab values across all groups.	Dysmenorrhea: placebo: –5.8(17.1); Relugolix: –15.4(18.1)/–19.8(20.4)/–29.5(17.5); Leuprorelin: –27.2(19.9).<br>Dyspareunia: placebo: –1.1(12.7); Relugolix: –3.5(10.9)/–3.6(11.6)/–0.9(12.0); Leuprorelin: –4.6(15.1).<br>Dose-response observed (pelvic pain, dysmenorrhea); Relugolix 40mg comparable to leuprorelin.	in relugolix 20/40 mg groups compared to placebo; comparable to leuprorelin. Almost all TEAEs were mild or moderate.	a, TEAEs, BMD mean % change from baseline: Moderate ⊕⊕⊕◯	by dose-response.
Miller CE et al. (2024) (28)	Phase 3, double-blind RCT	679 premenopausal women aged 18-49 with moderate-to-severe EAP	07.2017-08.2021	Elagolix 200 mg twice daily with AB therapy (1mg estradiol/0.5mg norethindrone acetate once daily) for 12 months (N=389→215) or elagolix 200 mg twice daily monothera	Placebo (N=193→106)	AbbVie Inc.	Low	Clinically meaningful pain reduction (reduction ≥0.92 for dysmenorrhea and ≥0.55 for NMPP on a 0–3 scale) with no increase in analgesic use at month 6: Dysmenorrhea: elagolix + AB therapy 62.8% vs	Mean Δ from Baseline (Elagolix+AB vs placebo):<br>Dysmenorrhea: M3: –1.54 vs –0.56 (p<0.001); M6: –1.64 vs –0.62 (p<0.001); M12: –1.73 vs –0.73 (p<0.001).<br>NMPP: M3: –0.65 vs –0.49 (p=0.004); M6: –0.78 vs –0.57 (p<0.001); M12: –0.86 vs –0.64 (p=0.002).	TEAEs: Elagolix+AB (73.8%) vs placebo (66.8%). Common: hot flushes (17.7%), nausea (13.1%), headache (11.8%).<br>SAEs: 14 vs 8 (0 deaths).<br>BMD (Δ LS mean %):<br>M6: Lumbar: –0.83% (p<0.05); Hip: –0.13% (NS); Neck: –0.06% (NS).<br>M12: Lumbar: –1.14% (p<0.05);	For dysmenorrhea and NMPP reduction (3, 6 and 12 months), Fatigue (PROMIS Fatigue T-score) (6 month), TEAEs, SAEs, BMD loss (6 and 12 months): High ⊕⊕⊕⊕	High Certainty: Large-scale, multicenter Phase 3 RCT. Maintained double-blind, placebo-controlled design for full 12 months, eliminating extension-phase RoB.

				py for 6 months and elagolix + AB therapy for the next 6 months (N=97→59)				placebo 23.7% (p< 0.001) NMPP: elagolix + AB therapy 51.3% vs placebo 36.8% (p< 0.001	Fatigue (PROMIS T-score, M6): –6.67 vs –4.71 (p<0.01).	Hip: –0.92% (p<0.05); Neck: –0.46% (NS). Elagolix+AB loss <1% (all sites, 12m). ELA mono (M6): –2.43% (lumbar).		Results show high precision and low attrition, providing robust evidence for efficacy and safety.
Simon J et al. (2023) (30)	36-month open-label extension study	215 premenopausal women (aged 18–49) with moderate-to-severe EAP completing 12-month period; transitioned to 200 mg elagolix + AB open-label extension	07.2017-08.2021	Elagolix 200 mg twice daily with AB therapy once daily (N=215→94) for 36 months	No control group	AbbVie Inc.	Moderate	BMD loss (Mean % Change from Baseline): Lumbar Spine: −0.77% (n=94); Total Hip: −0.36% (n=92); Femoral Neck: −1.39% (n=92)	Not numerically reported in the 36-month abstract; however, efficacy was described as sustained and consistent with earlier 12- and 24-month findings.	The safety profile (AEs and SAEs) remained consistent with previous 12- and 24-month reports, with no new safety signals identified up to 36 months of continuous treatment.	For BMD loss, AEs, SAEs, efficacy: Low ⊕⊕◯◯	Downgraded due to study design: ppen-label extension with no control group.
Harada T et al. (2024) (32)	Double-blind RCT	162 women aged ≥20 years with	08.2021 -	E4 15 mg + DRSP 3 mg taken for 24	Placebo (N =83→77)	Drug company grant (Fuji	Low	Change in VAS (pelvic pain):	Pain (NRS LSMD (E4/DRSP-Placebo)):	TEAEs: 69.6% (E4/DRSP) vs 51.8% (placebo)	For change in VAS score for	Downgraded for imprecisio

		endometriosis	11.2022	days followed by 4 days of placebo per cycle, for a total of 6 cycles (24 weeks) (N=79→75)		Pharma Co., Ltd.)		E4/DRSP (–31.1 mm) vs Placebo (–22.6 mm); Diff: –8.5 mm (p=0.028)	Dysmenorrhea (NRS)–1.63, Dyspareunia (NRS)–1.34, NMPP (NRS) –1.01; Rate Diff (E4/DRSP vs Placebo): Vol. Red. ≥50%: 31.3%, ≥30%: 40.6%; Fixed tenderness improv. 26.3%, Tenderness improv. 21.7%		the most severe pelvic pain, Dysmenorrhea, Dyspareunia, ≥50% and ≥30% reduction in ovarian endometrioma, NMPP, fixed tenderness: Moderate ⊕⊕⊕◯	n: the sample size (N=162) is limited.
Kobayashi T et al. (2024) (34)	Randomized, open-label, active-controlled, parallel-group clinical study	86 women aged 20-50 diagnosed with endometriosis	First patient enrolled on March 5, 2020	E4 15 mg + DRSP 3 mg, taken orally once daily for 24 active days followed by 4 hormone-free days, for 3 cycles (12 weeks) (N=45→44)	EE 20 µg/DRSP 3 mg (N=43→38)	Drug company grant (Fuji Pharma Co., Ltd.)	Low	Mean % change from baseline to week 12 in nAPCsr: E4/DRSP group: 41.5% (95% CI: 16.4 to 66.7) EE/DRSP group: 154.4% (95% CI: 127.7 to 181.2) Difference between groups: -112.9% (95% CI: -149.6 to -76.2); (p<0.05)	ΔCoagulation/Fibrinolysis Parameters (%) (E4/DRSP vs. EE/DRSP): D-dimers: –131.3% Free Protein S: +28.9% Free TFPI: +42.4 Prothrombin F1+2: –51.2% Factor VII: –23.2% Protein C Ag: –13.0%	NR	For mean % change from in nAPCsr, changes in coagulation and fibrinolysis parameters: Low ⊕⊕◯◯	Downgraded for serious imprecision (Phase II, small sample size N=86, wide CIs).

Rostami S et al. (2023) (37)	Triple-blind RCT	50 women aged 20–40 with established stage III/IV endometriosis infertility, who are candidates for ART	12.2021 - 05.2022	AST 6 mg per day for 12 weeks prior to ART (N28→25)	Placebo (N=29→25)	Tehran University of Medical Sciences	Low	Change in serum level of TAC 398.661 ± 57.686 in the AST group vs. 364.746 ± 51.569 in the placebo group (p= 0.004)	ART Outcomes (AST vs placebo): Oocytes retrieved: 14.6±7.8 vs 9.8±6.4 (p=0.043) Mature oocytes: 10.5±6.7 vs 6.7±4.3 (p=0.041) High-quality embryos: 4.5±2.4 vs 2.7±2.4 (p=0.024) Inflammatory Cytokines (pg/ml): IL-1β: 4.5±0.9 vs 6.9±0.8 (p<0.001 IL-6: 5.5±0.6 vs 5.1±0.7 (p=0.024 TNF-α: 2.5±0.5 vs 3.0±0.5 (p=0.038) Oxidative Stress Markers: MDA (µmol/L): 14.6±2.5 vs 15.9±1.5 (p=0.031) SOD (U/ml): 13.5±7.3 vs 9.0±5.2 (p=0.010) CAT (U/ml): 0.28±0.15 vs 0.24±0.13 (NS)	NR	For Change in serum level of TAC, number of oocytes retrieved, mature oocytes, high-quality embryos, Effect of AST on pro-inflammatory cytokines, Effect on oxidative stress marker: Low ⊕⊕◯◯	Evidence was downgraded due to serious imprecision stemming from the small sample size (N=50) and wide confidence intervals and limited statistical power.
Khodarahmian M et al. (2021) (42)	Parallel, double-blind exploratory RCT	34 infertile women (aged 18–37) with regular cycles and laparoscopically confirmed stage III–IV	05.2016 – 07.2017	400 mg resveratrol twice daily (800 mg/day) for 12–14 weeks (N=17→17)	Placebo twice daily for 12–14 weeks (N=17→17)	Tehran University of Medical Sciences	Low	Effects of resveratrol on VEGF and TNF-α expression in eutopic endometrium ↓ VEGF (mRNA p=0.017,	NR	NR	For effects on VEGF and TNF-α expression in eutopic endometrium: Low ⊕⊕◯◯	Downgraded for very serious imprecision (N=34). Study designated as "exploratory"; lack of reported

		endometriosis						protein p=0.012) ↓ TNF-α (mRNA p=0.018, protein p=0.019)				95% CIs (only p-values provided) prevents precise estimation of treatment effect. No serious RoB or indirectness concerns.
DiVasta AD et al. (2021) (43)	Double-blind, pilot RCT	9 premenopausal women aged 15–40 with surgically confirmed endometriosis and chronic pelvic pain	2016-2018	Cabergoline (0.5 mg, twice weekly) + placebo (daily) for 6 months (N=5→4)	NETA (5 mg/day) + placebo (twice weekly) for 6 months (N=4→3)	Marriott Daughters' Foundation	High	Pain scores (Brief Pain Inventory / VAS) at 6 months. Numerical data not reported as Mean ± SD due to pilot study design (n=7 completers). Authors reported trends: Cabergoline group showed a reduction in 'worst pain' score from 8.0 (baseline) to 3.0 (6 months). Statistical significance	Change in inflammatory and angiogenic pathway parameters - serum VEGF-R1 decreased with cabergoline (417.6 to 344.1 pg/ml) but increased with NETA (330.7 to 447.6 pg/ml)] serum IL-8 levels halved with cabergoline (9.5 to 4.5 pg/mL) but showed no significant change with NETA (4.8 to 4.9 pg/mL)	SAEs = 0. Cabergoline was associated with higher rates of nausea (4 vs 0) and constipation (3 vs 1), whereas NETA caused greater weight gain (+2.5 kg vs +1.3 kg).	For Pain scores, serum VEGF-R1, serum IL-8 levels: Very low ⊕◯◯◯	Downgraded due to serious RoB (pilot design, no between-group statistical analysis) and very serious imprecision (extremely small sample size, n=7). Results represent numerical trends only; baseline imbalances in biomarkers further

								between groups was not calculated.				limit reliability.
Sullender R et al. (2024) (53)	Single-site, double-blinded, cross-over pilot RCT	30 females aged 18-45 with menstrual cycles every 24-32 days with a surgical diagnosis of endometriosis or an endometrioma on imaging (2 groups: 7 participants - cycle of drug then placebo, 8 participants - cycle of placebo then drug)	2019-2023	Anakinra 100 mg, daily for three menstrual cycles and placebo for three menstrual cycles (in different order within the groups)	15 (group A: n=7 - cycle of drug then placebo, group B: n=8 - cycle of placebo then drug)	Swedish Orphan Biovitrum	High	Improvement in dysmenorrhea using the dysmenorrhea specific portion of the modified B&B questionnaire completed monthly at the end of each period (anakinra treatment cycles=1.4, placebo=1.6) and daily VAS (anakinra=37.5, placebo=42.6)	QoL (EHP-30, Anakinra vs placebo): Pain: 46.3 vs 49.4; Powerless: 54.5 vs 63.3; Emotional WB: 39.1 vs 44.7; Soc. Support: 45.1 vs 50.8; Self-image: 58.1 vs 66.7. Cycle Markers (Anakinra vs placebo): Bleeding (days): 5.0 vs 5.3; Cycle length (days): 29.3 vs 27.7. Inflammatory Biomarkers (Anakinra vs placebo): IL-1RA (Grp B): 5093 vs 8.7 (p=0.005); IL-6 (Grp A): 1.28 vs 1.70 (p=0.028)	NR	For improvement in dysmenorrhea, daily VAS, changes in QoL, inflammatory biomarkers: Very Low ⊕◯◯◯ For changes in menstrual cycle length: Low ⊕⊕◯◯	Certainty ranges from Low to Very Low. Most outcomes were downgraded for RoB due to the pilot nature of the study, the small sample size (n=15) and extreme imprecision - the sample size is well below the Optimal Information Size.

2PN: two-pronuclear; **aHR**: adjusted hazard ratio; **AB**: add-back therapy; **AEs**: adverse events; **Ag**: antigen; **AMH**: anti-Mullerian hormone; **ART**: assisted reproductive techniques; **AST**: astaxanthin; **B&B**: Biberoglu and Behrman scale; **BMD**: bone mineral density; **CA-125**: cancer antigen-125; **CAT**: catalase; **CES-D**: Center for Epidemiologic Studies Depression Scale; **COCP**: combined oral contraceptive pill; **COCs**: combined oral contraceptives; **CT**: combination therapy; **DNG**: dienogest; **DRSP**: drospirenone; **E2/NOMAC**: nomegestrol acetate; **E4**: estetrol; **EAP**: endometriosis-associated pain; **ECG**: electrocardiogram; **EE**: ethinyl estradiol; **EOT**: end of treatment; **GnRH**: gonadotropin-releasing hormone; **GRADE:** Grading of Recommendations Assessment, Development, and Evaluation; **LSM**: least squares mean; **LSMD**: least squares mean

difference; **M**: month(s); **MDA**: malondialdehyde (lipid peroxidation marker); **nAPCsr**: normalized activated protein C sensitivity ratio; **NETA**: norethindrone acetate; **NMPP**: non-menstrual pelvic pain; **NRS**: Numerical Rating Scale; **NR**: not reported; **NS**: not significant; **OCPs**: oral contraceptive pills; **QoL**: quality of life; **RCT**: Randomized Controlled Trial; **RoB**: Risk of Bias; **SAEs**: serious adverse events; **SOD**: superoxide dismutase (protective enzyme); **STAI:** State Trait Anxiety Inventory; **TAC**: total antioxidant capacity; **TEAEs**: treatment-emergent adverse events; **TNF-α**: tumor necrosis factor-alpha; **VAS**: Visual Analogue Scale; **VEGF**: vascular endothelial growth factor; **VTE**: venous thromboembolism; **w**: week(s); **y**: year(s).
