# Supplementary material for: Current Evidence and Emerging Directions in Endometriosis Pharmacotherapy: A Systematic Review of Recent Advances
Source: J Clin Med. 2026 Sep 17;15(18):7238. doi: 10.3390/jcm15187238 (PMC13607422; doi:10.3390/jcm15187238)
Supplement: Supplementary file 1 [file jcm-15-07238-s001.zip › Table S2 GRADEtable.pdf]

Table S2 GRADE Summary of Findings

Outcome	Design	Limitations (*RoB score)*	Indirectness of patients, intervention and comparator	Inconsistency	Imprecision	Other considerations	Quality of evidence
Niakan et al. (2021) (9)							
For pelvic pain, dyspareunia and QoL	Double-blind RCT	Some concerns	0	N/A	-1	0	Moderate ⊕⊕⊕◯
Yurtkal et al. (2024) (10)							
For endometriosis pain	Prospective cohort study	Moderate	-1	N/A	-1	0	Very low ⊕◯◯◯
Caruso s et al. (2022) (11)							
For chronic pelvic pain, QoL and sexual function	Randomized, comparative study	Moderate	-1	N/A	-1	0	Low ⊕⊕◯◯
Alithan et al. (2025) (13)							
For reduction in endometrioma size and pain	Retrospective cohort study	Moderate	-1	N/A	-1	0	Very low ⊕◯◯◯
La Torre et al. (2024) (14)							
For long-term pain relief, safety and tolerability and treatment persistence	Cohort study	Low	-1	N/A	-1	0	Very low ⊕◯◯◯
Park Y et al. (2025) (15)							
For reduction of menstrual pain, of the size of ovarian endometrioma,	Prospective study	Serious	0	N/A	-1	0	Very Low ⊕◯◯◯
For changes in psychiatric variables and risk factors associated with depressive symptoms	Prospective study	Serious	-1	N/A	-1	0	Very Low ⊕◯◯◯
Kim et al. (2021) (17)							
For BMD and recurrence rate	Retrospective cohort study	Moderate	-1	N/A	-1	0	Very Low ⊕◯◯◯
Jhang H et al. (2025) (18)							
For risk of breast cancer, endometrial cancer, tubo-ovarian cancer	Retrospective cohort study	Moderate	0	N/A	-1	0	Very Low ⊕◯◯◯
Barra et al. (2020) (20)							
For clinical pregnancy rate, number of oocytes and implantation rate	Retrospective cohort study	Moderate	0	N/A	-1	0	Very Low ⊕◯◯◯
Tamura et al. (2019) (21)							
For pregnancy rate for	Prospective cohort study	Moderate	0	N/A	-1	0	Very Low ⊕◯◯◯

Table S2 GRADE Summary of Findings

transfer, numbers of oocyted recieved and cumulative pregnancy rate							
Mehdizadeh K et al. (2022) (60)							
For change in pain scores, QoL	Double-blind, pilot RCT	Some concerns	0	N/A	-1	0	Moderate ⊕⊕⊕◯
For pain Rrcurrence			0	N/A	-2	0	Low ⊕⊕◯◯
Ma Y et al. (2024) (12)							
For incidence of irregular vaginal bleeding	Retrospective cohort study	Serious	-1	N/A	-1	0	Very low ⊕◯◯◯
For pelvic pain, CA125 levels, recurrence rate			0	N/A	-1	0	Very low ⊕◯◯◯
Giudice LC et al. (2022) SPIRIT 1 (24)							
For dysmenorrhea, NMPP, EHP-30 pain domain, overall pelvic pain, dyspareunia, analgesic-free and opioid-free status, TEAEs, SAEs and BMD loss:	Phase 3, double-blind RCT	Low	0	N/A	0	0	High ⊕⊕⊕⊕
Giudice LC et al. (2022) SPIRIT 2 (24)							
For dysmenorrhea, NMPP, overall pelvic pain. dyspareunia reduction, EHP-30 pain domain, analgesic-free and opioid-free status, TEAEs, SAEs and BMD loss	Phase 3, double-blind RCT	Low	0	N/A	0	0	High ⊕⊕⊕⊕
Becker CM et al. (2024) (25)							
For dysmenorrhea, NMPP, overall pelvic pain and dyspareunia reduction, EHP-30 pain domain, analgesic-free and opioid-free status, TEAEs, SAEs and BMD	Long-term open-label extension study	Moderate	0	N/A	0	0	Low ⊕⊕◯◯
Osuga Y et al. (2021)a (26)							

Table S2 GRADE Summary of Findings

For dyspareunia and SAEs BMD (mean % change from baseline), AEs	Phase 2, double-blind RCT	Low	0	N/A	-1	0	Moderate ⊕⊕⊕◯
For mean change in VAS (pelvic pain) and dysmenorrhea			0	N/A	-1	1	High ⊕⊕⊕⊕
Osuga Y et al. (2021)b (27)							
For mean change in VAS (pelvic pain, dysmenorrhea) (24 weeks)	Phase 2, open-label, parallel-group extension RCT	Some concerns	0	N/A	-1	1	High ⊕⊕⊕⊕
For dyspareunia, TEAEs, BMD mean % change from baseline			0	N/A	-1	0	Moderate ⊕⊕⊕◯
Miller CE et al. (2024) (28)							
For dysmenorrhea and NMPP reduction (3, 6 and 12 months), Fatigue (PROMIS Fatigue T-score) (6 month), TEAEs, SAEs, BMD loss (6 and 12 months):	Phase 3, double-blind RCT	Low	0	N/A	0	0	High ⊕⊕⊕⊕
Simon J et al. (2023) (30)							
For BMD loss, efficacy, AEs, SAEs	36-month open-label extension study	Moderate	0	N/A	0	0	Low ⊕⊕◯◯
Harada T et al. (2024) (32)							
For change in VAS score for the most severe pelvic pain, Dysmenorrhea, Dyspareunia, ≥50% and ≥30% reduction in ovarian endometrioma, fixed tenderness	Double-blind RCT	Low	0	N/A	-1	0	Moderate ⊕⊕⊕◯
Kobayashi T et al. (2024) (34)							
For mean % change from in nAPCsr, changes in	Randomized, open-label, active-controlled,	Low	0	N/A	-2	0	Low ⊕⊕◯◯

Table S2 GRADE Summary of Findings

coagulation and fibrinolysis parameters	parallel-group clinical study						
Rostami S et al. (2023) (37)							
For Change in serum level of TAC, number of oocytes retrieved, mature oocytes, high-quality embryos, Effect of AST on pro-inflammatory cytokines, effect on oxidative stress marker	Triple-blind RCT	Low	0	N/A	-2	0	Low ⊕⊕◯◯
Khodarahmian M et al. (2021) (42)							
For effects on VEGF and TNF-α expression in eutopic endometrium	Parallel, double-blind exploratory RCT	Low	0	N/A	-2	0	Low ⊕⊕◯◯
DiVasta AD et al. (2021) (43)							
For Pain scores, serum VEGF-R1, serum IL-8 levels	Double-blind, pilot RCT	High	0	N/A	-2	0	Very low ⊕◯◯◯
Sullender R et al. (2024) (53)							
For improvement in dysmenorrhea, daily VAS, changes in QoL, inflammatory biomarkers	Single-site, double-blinded, cross-over pilot RCT	High	0	N/A	-2	0	Very Low ⊕◯◯◯
For changes in menstrual cycle length:				N/A	-2	0	Low ⊕⊕◯◯
